# Targeted Deletion of *Nrf2* Reduces Urethane-Induced Lung Tumor Development in Mice

**DOI:** 10.1371/journal.pone.0026590

**Published:** 2011-10-21

**Authors:** Alison K. Bauer, Hye-Youn Cho, Laura Miller-DeGraff, Christopher Walker, Katherine Helms, Jennifer Fostel, Masayuki Yamamoto, Steven R. Kleeberger

**Affiliations:** 1 Department of Pathobiology and Diagnostic Investigation, College of Veterinary Medicine, Michigan State University, East Lansing, Michigan , United States of America; 2 Laboratory of Respiratory Biology, National Institute of Environmental Health Sciences, National Institutes of Health, Research Triangle Park, North Carolina, United States of America; 3 Division of Medical Biochemistry, Graduate School of Medicine, Tohoku University, Sendai, Japan; Enzo Life Sciences, Inc., United States of America

## Abstract

Nrf2 is a key transcription factor that regulates cellular redox and defense responses. However, permanent Nrf2 activation in human lung carcinomas promotes pulmonary malignancy and chemoresistance. We tested the hypothesis that *Nrf2* has cell survival properties and lack of *Nrf2* suppresses chemically-induced pulmonary neoplasia by treating *Nrf2* ^+/+^ and *Nrf2* ^-/-^ mice with urethane. Airway inflammation and injury were assessed by bronchoalveolar lavage analyses and histopathology, and lung tumors were analyzed by gross and histologic analysis. We used transcriptomics to assess Nrf2-dependent changes in pulmonary gene transcripts at multiple stages of neoplasia. Lung hyperpermeability, cell death and apoptosis, and inflammatory cell infiltration were significantly higher in *Nrf2* ^-/-^ mice compared to *Nrf2* ^+/+^ mice 9 and 11 wk after urethane. Significantly fewer lung adenomas were found in *Nrf2* ^-/-^ mice than in *Nrf2* ^+/+^ mice at 12 and 22 wk. Nrf2 modulated expression of genes involved cell-cell signaling, glutathione metabolism and oxidative stress response, and immune responses during early stage neoplasia. In lung tumors, Nrf2-altered genes had roles in transcriptional regulation of cell cycle and proliferation, carcinogenesis, organismal injury and abnormalities, xenobiotic metabolism, and cell-cell signaling genes. Collectively, *Nrf2* deficiency decreased susceptibility to urethane-induced lung tumorigenesis in mice. Cell survival properties of Nrf2 were supported, at least in part, by reduced early death of initiated cells and heightened advantage for tumor cell expansion in *Nrf2* ^+/+^ mice relative to *Nrf2* ^-/-^ mice. Our results were consistent with the concept that Nrf2 over-activation is an adaptive response of cancer conferring resistance to anti-cancer drugs and promoting malignancy.

## Introduction

Lung cancer is the leading cause of cancer mortality worldwide, thus creating an enormous public health burden [1]. Adenocarcinoma (AC), the predominant subtype of non-small cell lung carcinoma (NSCLC), is the most prevalent NSCLC among smokers and is the only lung cancer found in non-smokers [2]. Unfortunately due to the lack of useful biomarkers, AC is rarely detectable until advanced stages of the disease, which makes it one of the most clinically intractable of lung cancers [3].

In mice, urethane induces lung ACs and the time frame for development of hyperplasias, adenomas, and ACs is well characterized and accepted as a model for human lung AC [4], [5], [6]. Histopathologically, murine ACs are indistinguishable from human AC [4], [7]. The progenitor cells for AC development include type II pneumocytes and Clara cells [4], [7]. ACs are associated with mutations in *Kras* oncogene and tumor suppressor genes such as *Tp53* in humans and mice.

Nuclear factor, erythroid derived 2, like 2 (Nrf2), is a ubiquitous key modulator of cellular defense against oxidative stress and inflammation. Nrf2 binds to a *cis*-acting antioxidant response element (ARE) to induce transcription of multiple cytoprotective proteins including phase 2 detoxifying enzymes, drug efflux pumps, and reactive oxygen species (ROS) scavengers [8]. Kelch-like ECH-associated protein 1 (Keap1) suppresses Nrf2 to maintain homeostasis of Nrf2-ARE responsiveness by sequestering and driving proteasomal degradation of cytoplasmic Nrf2 and by shuttling nuclear Nrf2 to the cytoplasm. Stimuli which dissociate Nrf2 from Keap1 to activate Nrf2 include pro-oxidants, antioxidants, and chemopreventive agents. In multiple models of pulmonary non-neoplastic inflammatory and oxidant diseases, exacerbated injury, inflammation, and oxidant stress were found in *Nrf2* deficient mice (*Nrf2^-/-^*) compared to wild type (*Nrf2^+/+^*) mice [8], [9], [10].

Importantly, recent studies have revealed that somatic mutations in functional domains or epigenetic changes (methylation) in CpG islands of KEAP1 which decrease KEAP1 function are associated with increased risk of human lung neoplasia and chemoresistance [11], [12], [13], [14]. Further, siRNA silencing of NRF2 in these neoplastic cells inhibited their growth and recovered chemotherapeutic sensitivity [13], [15]. These studies suggested that lung cancer cells with ’permanent’ NRF2-ARE activation due to weakened KEAP1 acquired multiple advantages for proliferation and resistance to chemotherapy promoting malignancy. However, molecular mechanisms explaining this potential advantage for cancer cell growth have not been fully elucidated. In the current study, we tested the hypothesis that Nrf2 has a cell survival role in chemical-induced pulmonary neoplasia using *Nrf2^+/+^* and *Nrf2^-/-^* mice. Using bioinformatic tools for transcriptome analysis, we defined potential downstream effector mechanisms of Nrf2-directed lung tumorigenesis.

## Results

### Differential urethane sensitivity during pre-neoplastic stage

#### Pulmonary injury

**Figure 1:**
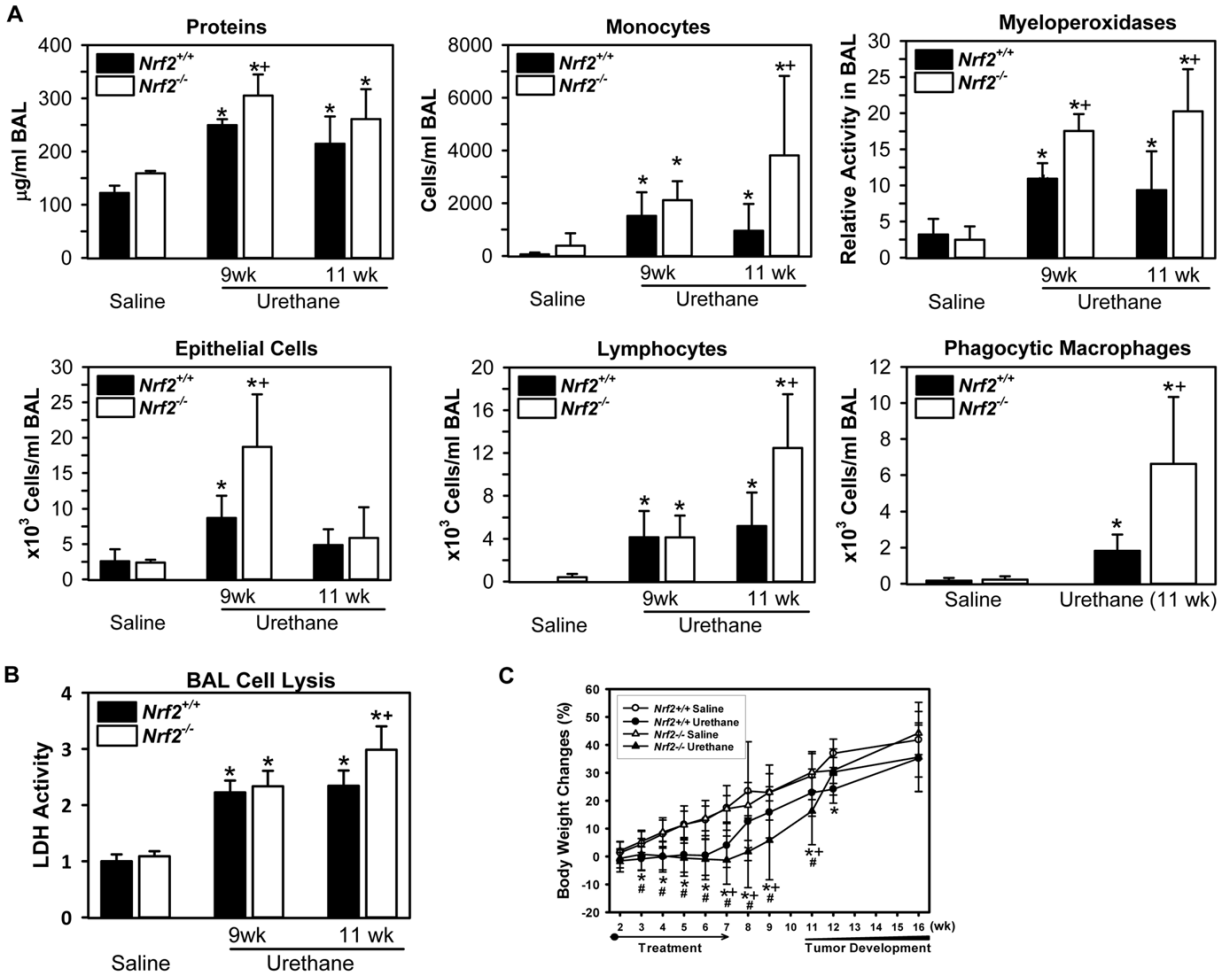
Bronchoalveolar lavage analysis (BAL) and body weight changes at a pre-neoplastic stage. (A) BAL analysis found significantly higher concentration of total protein and numbers of monocytes, lymphocytes, phagocytic macrophages, and epithelial cells in *Nrf2^-/-^* mice at 9 and/or 11 wk after urethane treatment. Neutrophilic myeloperoxidase (MPO) activity in BAL fluids was also significantly higher in *Nrf2^-/-^* mice than in *Nrf2^+/+^* mice. Mean±SD are presented (n = 3/group for vehicle, n = 5−9/group for urethane). *, p<0.05 vs. genotype-matched controls. +, p<0.05 vs. treatment-matched *Nrf2^+/+^* mice. (B) Necrotic lung cell lysis and death was assessed by lactate dehydrogenase (LDH) activity in aliquots of BAL fluids using a colorimetric assay. Mean±SD are presented (n = 3/group for vehicle, n = 5−9/group for urethane). *, p<0.05 vs. genotype-matched controls. +, p<0.05 vs. treatment-matched *Nrf2^+/+^* mice. (C) Percent whole body weight changes monitored during and after saline or urethane treatment. Mean±SD are presented (n = 9−24 in vehicle groups, n = 64−82 in urethane groups). *, p<0.05 *Nrf2^+/+^* saline-treated vs. urethane-treated mice (*p*<0.05). #, p<0.05 *Nrf2* ^-/-^ saline-treated vs. urethane-treated mice. +, p<0.05 urethane-treated *Nrf2^+/+^* mice vs. urethane-treated *Nrf2^-/-^* mice (*p*<0.05).

Microscopic analysis of bronchoalveolar lavage (BAL) cells indicated pulmonary cytotoxicity including lysis at 9–11 wk after initial urethane injection in *Nrf2* ^-/-^ and *Nrf2* ^+/+^ mice. Increases in mean numbers of BAL epithelial cells and leukocytes, as well as total protein concentration, were significantly greater in *Nrf2* ^-/-^ than *Nrf2* ^+/+^ mice after 9–11 wk (Figure 1-A). By 11 wk, BAL cell aggregation was mostly resolved in both strains, but cell lysis remained in *Nrf2* ^-/-^ mice with concurrent manifestation of significantly higher large atypical macrophages showing phagocytosis or necrosis-like features than in *Nrf2* ^+/+^ mice (Figure 1A; Figure S1). Significantly greater membrane rupture was also found in BAL cells from *Nrf2* ^-/-^ mice than *Nrf2* ^+/+^ mice (Figure S1). At 9 wk, BAL cell clustering accompanied cytolysis, and this feature was markedly greater in *Nrf2* ^-/-^ mice compared to *Nrf2* ^+/+^ mice (Figure S1). This observation is assumed to be associated with excess viscous secretion such as mucus as suggested by aggregation of cells with secreted mucus in Alcian Blue (pH 2.5)/periodic acid-Schiff (AB/PAS)-stained lungs (Figure S1), or by procoagulant activity due to fibrin deposition in mononuclear phagocytes which occurs during lung inflammation [16], [17]. Infiltrated neutrophils were obvious but not quantifiable due to extensive clustering and lysis of BAL cells in *Nrf2* ^-/-^ mice; consistent with this observation, neutrophil myeloperoxidase (MPO) activity was significantly higher in *Nrf2* ^-/-^ mice than in *Nrf2* ^+/+^ mice treated with urethane (Figure 1A). Urethane-induced increase in lactate dehydrogenase (LDH) activity, a quantitative indicator of necrotic cell lysis, was also significantly greater in BAL returns from *Nrf2* ^-/-^ compared to *Nrf2* ^+/+^ mice (Figure 1B).

#### Body weight (BW) changes

Urethane restrained BW gain during the injection period (1–11 wk) in both genotypes relative to saline injection (Figure 1C). However, a lower BW was observed in *Nrf2^-/-^* mice than in *Nrf2^+/+^* mice during the inflammatory period from 7–11 wk (Figure 1C).

### Differential responses in early neoplastic stage

#### Apoptosis and proliferation

**Figure 2:**
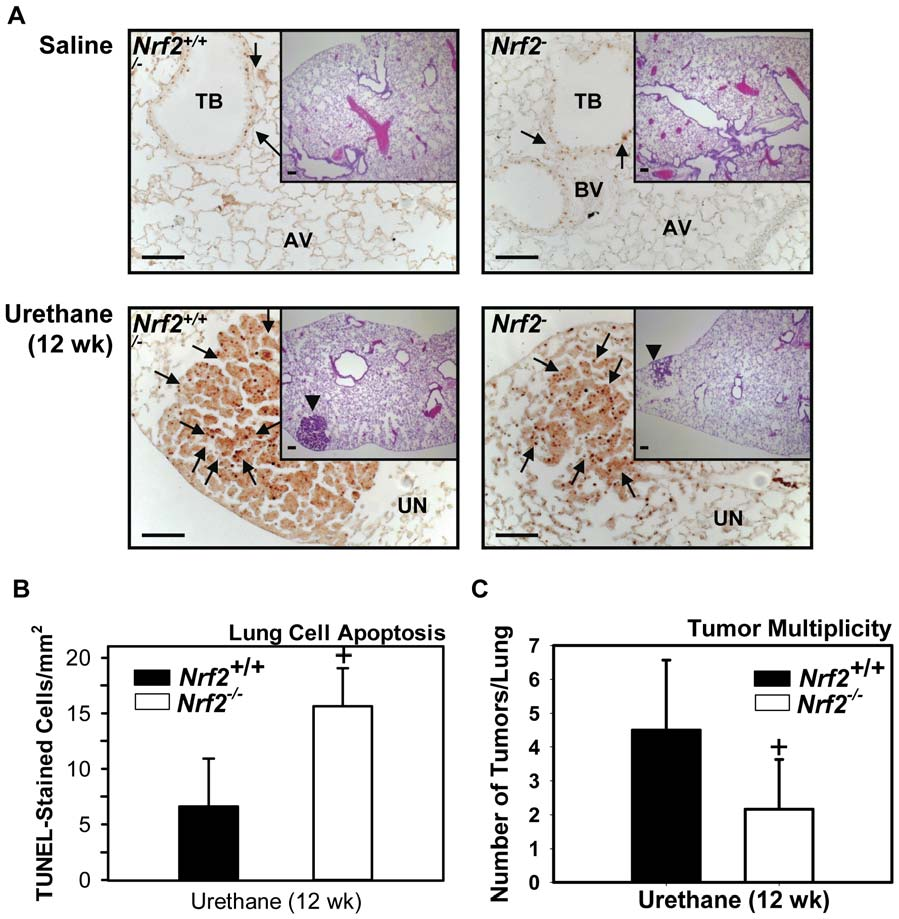
Early stage tumorigenesis at 12 wk. (A) H&E-staining demonstrate pulmonary hyperplastic regions and early tumor development (arrow heads) 12 wk after the first urethane injection. Greater tumor cell proliferation in sporadically small adenomatous regions was found in *Nrf2^+/+^* mice than *Nrf2^-/-^* mice as indicated by denser proliferating cell nuclear antigen (PCNA) localization in relatively more advanced tumors (inset). Representative light photomicrographs showing intermediate magnitude of pathology for each treatment group are presented (n = 3−8/group for H&E, n = 3/group for PCNA). AV, alveoli; TB, terminal bronchiole; BV, blood vessel; UN, uninvolved region. Arrow heads, tumor; arrows, PCNA-positive nuclei. Bars indicate 100 µm. (B) Numerous apoptotic airway cells in *Nrf2^-/-^* mice were identified by TUNEL assay on paraffin-embedded lung tissue sections. TUNEL-positive nuclei of epithelial, endothelial, and smooth muscle cells on proximal lung sections were counted and normalized to airway surface (mm^2^) using digital image processing software. Few TUNEL-stained cells were found in vehicle control mice. Mean±SD are presented (n = 3/group). +, p<0.05 vs. urethane-treated *Nrf2^+/+^* mice. (C) Differential early tumor formation between *Nrf2^+/+^* and *Nrf2^-/-^* mice. Average number of tumors (≥200 µm) per whole lung from each mouse was assessed in serial sections of paraffin-embedded lungs fixed with 10% NBF. Mean±SD are presented (n = 13−14/group). +, p<0.05 vs. urethane-treated *Nrf2^+/+^* mice.

Immunohistochemical detection of proliferating cell nuclear antigen (PCNA, a cell proliferation marker [18]) at 12 wk demonstrated that PCNA-positive cells in G1/S phase were localized extensively in small adenomas, focal alveolar hyperplastic lesions, injured perivascular and peribronchial regions, and nodular lymphoid aggregates in adventitia of blood vessels. Compared to *Nrf2* ^-/-^, more abundant and intense localization of PCNA were found in small adenomatous regions of *Nrf2* ^+/+^ mice (Figure 2A). PCNA was sporadic in conducting airway epithelium of saline controls (Figure 2A). Consistent with differential BAL cell necrosis at 9–11 wk, the number of apoptotic lung cells determined by terminal deoxynucleotidyl transferase-mediated dUTP nick-end labeling (TUNEL) in proximal pulmonary sections was more highly elevated in *Nrf2* ^-/-^ mice compared to *Nrf2* ^+/+^ mice at 12 wk (Figure 2B). TUNEL-positive cells included epithelial, endothelial, and smooth muscle cells. Few apoptotic cells were found in lungs of vehicle control mice.

#### Early adenoma formation

Lung tumors were evident and countable (>200 µm) from 12 wk following initiation of urethane treatment. The numbers of sporadic adenomas were significantly lower (2-fold) in *Nrf2* ^-/-^ mice compared to *Nrf2* ^+/+^ mice (Figure 2C).

### Differential tumorigenesis at 22 wk

**Figure 3:**
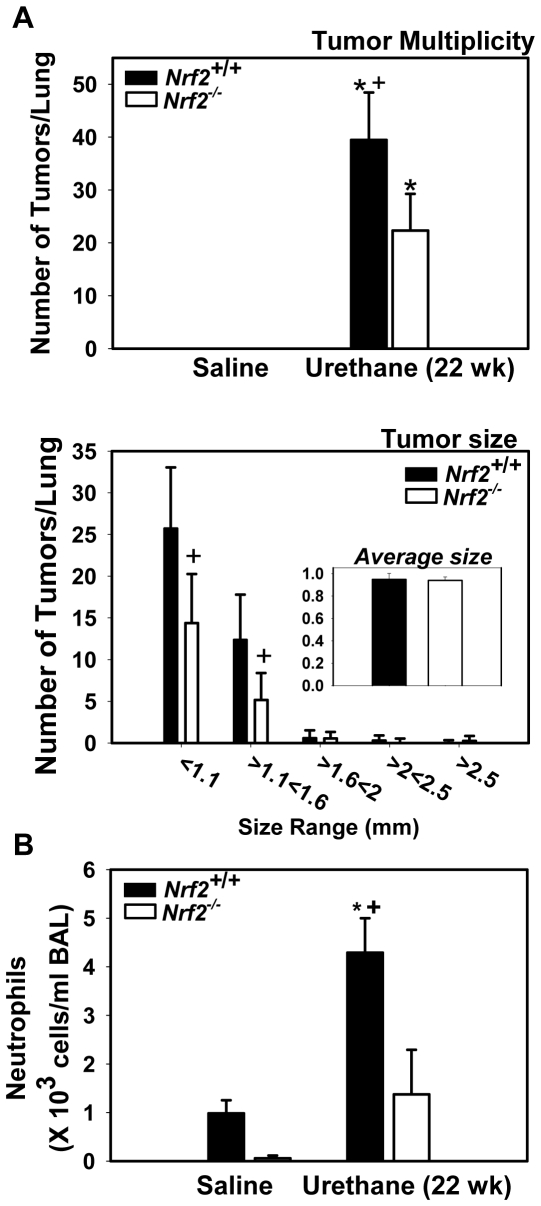
Adenoma development and persistent lung inflammation at 22 wk. (A) Average number of adenomas per whole lung from each animal (tumor multiplicity) and number of tumors per size ranges and average size (mm) of individual tumors were assessed in lungs fixed with Tellyesniczky's fixative. Mean±SD are presented (n = 4−5/group saline; n = 14/group for tumor multiplicity; n = 14−20/group for tumor size). This study was repeated once with similar n and responses determined. *, p<0.05 vs. genotype-matched controls. +, p<0.05 vs. urethane-treated *Nrf2^+/+^* mice. (B) Bronchoalveolar lavage (BAL) analysis of the number of neutrophils in *Nrf2^+/+^* and *Nrf2^-/-^* mice at 22 wk after urethane treatment. Mean±SD are presented (n = 7−10/group urethane treated and n = 3−5/group for saline). *, p<0.05 vs. genotype-matched controls. +, p<0.05 vs. urethane-treated *Nrf2^+/+^* mice.

Multiplicity (∼43%) of small (≤1.6 mm) adenomas was significantly reduced in *Nrf2* ^-/-^ mice compared to *Nrf2* ^+/+^ mice at 22 wk after urethane treatment (Figure 3A). However, no differences were found in overall tumor size or tumor morphology between genotypes. Large tumors were located primarily at the lung pleural surface, while small developing tumors were more frequent in internal regions. While no specific differences were observed in other BAL phenotypes, the numbers of neutrophils were significantly lower in *Nrf2* ^-/-^ mice compared to *Nrf2* ^+/+^ mice at 22 wk (Figure 3B).

### Nrf2 activation in tumor-bearing lungs

**Figure 4:**
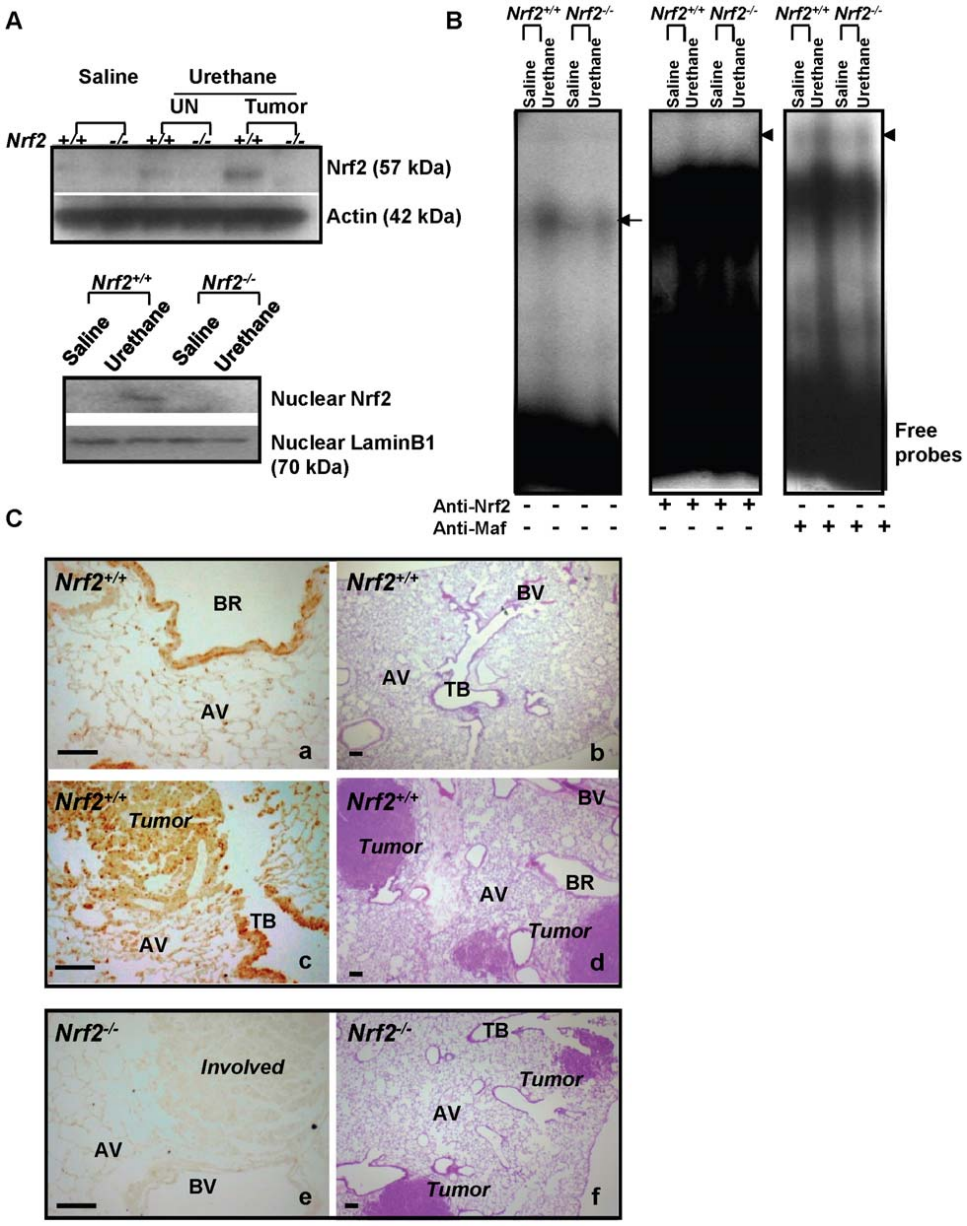
Pulmonary Nrf2 expression and activation caused by urethane. (A) Western blot analysis of total Nrf2 protein in uninvolved (UN) and tumor tissues compared to saline control in lung at 22 wk (57 kDa determined). Nuclear translocation of Nrf2 in urethane-treated lungs at 22 wk was determined by Western blot analysis in whole lung nuclear extracts (10 µg). Lamin B1 level was determined as an internal control. (B) Binding activity of nuclear protein (5 µg) to [γ^32^P]ATP end-labeled oligonucleotide probe containing ARE consensus sequence was determined. Gel shift analysis demonstrated increased total ARE binding activity (arrows) of lung nuclear proteins after urethane treatment in *Nrf2^+/+^* mice. Supershifted bands (arrow head) indicate specific binding activity of nuclear Nrf2 or small Maf on ARE determined by addition of anti-Nrf2 or –Maf (F/G/K) antibodies to the reaction. Representative digitized bands of Western blotting and gel shift analysis (n = 2/group) are presented. (C) Immunohistochemistry for Nrf2 in saline- (a) or urethane-treated (c,e) tissue sections. Greater localization of Nrf2 proteins (brown dots) in growing adenomas and in conducting airway and alveolar epithelial cells was found in *Nrf2^+/+^* mice. Nrf2 was located mainly in airway epithelial cells of vehicle control lungs. Lung sections from *Nrf2^-/-^* mice (e) are shown as a negative control for Nrf2. H&E-stained lower magnification of lung sections treated with vehicle (b) or urethane (d,f) are depicted for general histology. Representative images showing intermediate degree of Nrf2 staining for each treatment group are presented (n = 3/group). AV = alveoli, BR = bronchi or bronchiole, TB = terminal bronchiole, BV = blood vessel, Bar = 100 µm.

Compared to saline controls at 22 wk, lung Nrf2 protein was increased in tumor tissues and in remaining uninvolved lung tissues (UN: adjacent, non-tumor bearing regions) of *Nrf2* ^+/+^ mice (Figure 4A). Nuclear levels of Nrf2 (Figure 4A) and total ARE binding activity of nuclear proteins (Figure 4B) were also higher in tumor-bearing lungs. Specific binding activities of Nrf2 and its dimerization partner small Maf on ARE were also heightened by urethane (Figure 4B). Abundant Nrf2 was located throughout the epithelium lining bronchial/bronchiolar and alveolar airways in control mice (Figure 4Ca). At 22 wk after urethane, the Nrf2-bearing cell populations were apparent in hyperplastic foci and growing adenomas in addition to their increase in the airway epithelium (Figure 4Cc).

### Gene expression profiling during urethane-induced tumorigenesis

#### Transcriptomics in early tumorigenesis of Nrf2^+/+^ mice (12 wks)

**Figure 5:**
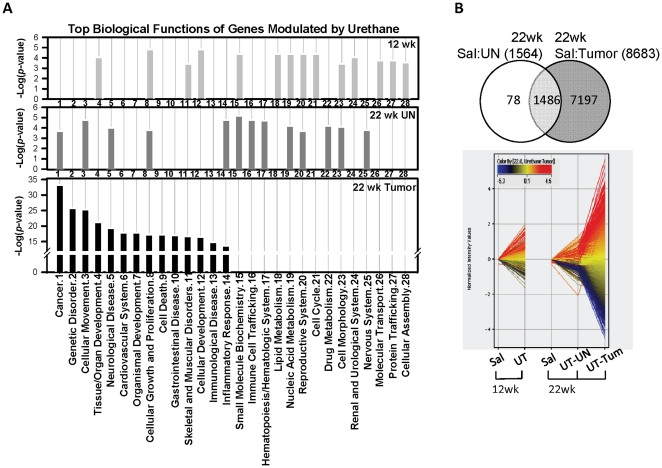
Lung gene expression profiles during urethane-induced tumorigenesis. (A) Urethane effects on gene expression during lung tumorigenesis were compared by biological function and disorder categories of genes significantly changed in pre-/early-neoplastic stage at 12 wk (top), in uninvolved tissues (UN) at 22 wk (middle), and in tumors at 22 wk (bottom) using Ingenuity Pathway Analysis (IPA). Top-ranked categories are depicted against -log(*p*) determined by IPA. (B) Among the lung gene transcripts significantly (*p*<0.05) changed in uninvolved (UN, n = 1564) and tumor (n = 8683) tissues compared to saline-treated lung tissues, Venn diagram analysis identified genes changed in either tissue or in common at 22 wk after the initial urethane treatment (top). Expression profiles of all tumor genes (n = 8683, 22 wk UT-Tum) are depicted in pre-/early neoplastic microenvironment (12 wk) and in UN tissues of tumor-bearing lungs at 22 wk (bottom). Color bar indicates expression intensity of individual transcripts normalized to their time-matched saline control levels expressed in yellow (yellow to blue, down-regulation; yellow to red, up-regulation). Y axis = log_2_ (normalized average intensity), Sal = saline-treated whole lung, UT = urethane-treated (whole lung for 12 wk, UN or Tum for 22 wk), UN = uninvolved tissue, Tum = tumor tissue.

To identify potential mechanisms of urethane-induced tumorigenesis, we then used trancriptomics of tumor and uninvolved tissue to identify differentiating gene networks. Significant changes in 6519 lung gene transcripts were found in *Nrf2^+/+^* mice relative to saline controls in 12 wk, and expression changes were mostly 2-fold or lower (Table S1; n = 3 mice/group). Functional classification by Ingenuity Pathway Analysis (IPA) determined that lung genes changed during early tumorigenesis have roles in cellular growth and proliferation, cell and tissue development, cell cycle and cellular assembly, small molecule biochemistry, lipid metabolism, and molecular transport (Figure 5A, top panel; Table S1).

#### Tumor gene expression profiles in Nrf2^+/+^ mice (22 wk)

Among genes significantly changed over saline controls at 22 wk in *Nrf2^+/+^* mice (8683 genes), 83% were changed significantly only in tumors (Figure 5-B; n = 3 mice/group). Expression kinetic profiles of genes modulated in tumor tissues (22 wk) were similar to their kinetics during early tumorigenesis (12 wk) and in the microenvironment of tumors (i.e., UN) at 22 wk (Figure 5B). Genes changed by urethane (mostly ≤2-fold) in UN tissues played roles mainly in small molecule biochemistry, immune cell trafficking, and cellular movement (Figure 5A, middle panel). A small number of genes (n = 78) associated with signal transduction, transcriptional regulation, ubiquitin-dependent protein catabolism, and muscle contraction were changed in UN, but not in tumors at 22 wk. Individual genes that demonstrated the greatest fold induction in the tumors included transmembrane protein 27 (*Tmem27*, 51-fold), BCL12-like 15 (*Bcl2l15*, 48-fold), and claudin 2 (*Cldn2*, 43-fold). In contrast, genes suppressed the most in the tumors included carbonic anhydrase 3 (*Car3*, -92-fold), myosin light polypeptide 7, regulatory (*Myl7*, -87-fold), troponin T2, cardiac (*Tnnt2*, -85-fold), actin, alpha, cardiac muscle 1 (*Actc1*, -52-fold), and cytochrome P450, family 2, subfamily a, polypeptide 4 (*Cyp2a4*, -36-fold). Also changes in numerous families of gene clusters were evident, which included ankyrin and ankyrin repeat domain, Rho GTPase activating proteins, chemokine (C-C motif) or (C-X-C motif) ligand and receptors, cell cycle protein families, C-type lectin domain family, *Cyp* family, fibroblast growth factors and receptors, gap junction protein (*Gj*) family, and G protein-coupled receptors. Genes changed more than 2-fold in tumors (n = 3461, up-regulated 1233 and down-regulated 2228 genes) were associated functionally in networks of genetic/skeletal and muscular/developmental disorders, cellular and hematological system development, hematopoiesis, cell cycle/growth, and cancer (Figure S2; Table S2). Genes involved in cellular growth and proliferation were modulated significantly by urethane regardless of the times (12 and 22 wk) and tissues (UN, tumors) during carcinogenesis, while genes associated with cancer (e.g., *Bcl6*, *Peg3*), cellular movement (e.g., *Prdx1*, *Cadm1*), neurological disease (e.g., *Cxcl1. Aff2*), and inflammatory response (e.g., *Itga4*, *Pla2g7*) were significantly changed in all the tissue types after tumor development at 22 wk (Figure 5A, lower panel). Genes for small molecular biochemistry (oxidation, metabolism, and transport reactions of acids, vitamins, and hormones) and lipid and nucleic acid metabolisms (e.g., *Cyp1a1*, *Slco1a5*, *Fabp1*) and cell morphology (e.g., *Cdkn1a*, *Cxcl9*) were modulated in common during the early tumorigenesis stage (12 wk) and in UN tissues at 22 wk (Figure 5A) suggesting similar molecular events are taking place in early neoplasia and in the tumor microenvironment.

#### Nrf2-dependent transcriptomics in early tumorigenesis (12 wk)

**Figure 6:**
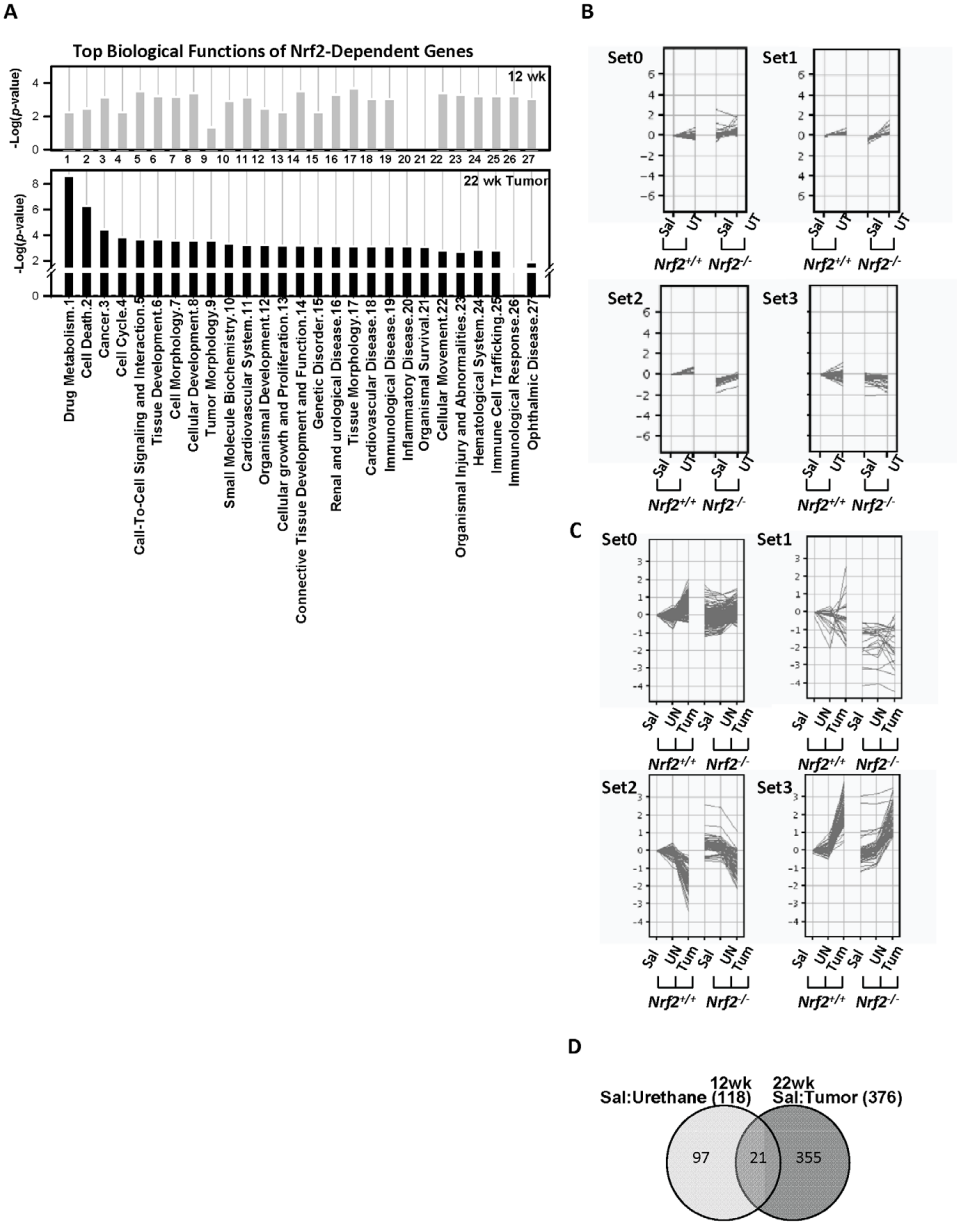
Effect of *Nrf2* deletion on gene expression during lung tumorigenesis. (A) Biological function and disorder categories of the Nrf2-dependent genes (i.e. differentially expressed between *Nrf2^+/+^* and *Nrf2^-/-^* mice) significantly changed in pre-/early neoplastic stage at 12 wk (top) and in tumors at 22 wk (bottom) were analysis by Ingenuity Pathway Analysis (IPA). Top-ranked [-log(*p*)] categories are depicted and compared between the two tumorigenesis stages. (B) A total of 118 gene transcripts varied significantly (*p*<0.05) between *Nrf2^+/+^* and *Nrf2^-/-^* mice 12 wk after urethane treatment were grouped into 4 expression profiles (set 0–3). Expression level of each transcript was normalized to corresponding *Nrf2^+/+^* saline controls and indicated as relative log ratio [log_2_ (normalized average intensity)]. Genes are listed in Dataset 1 and in Table 1. Sal = saline-treated lung, UT = urethane-treated lung. (C) Lung tumor genes (n = 376) significantly (*p*<0.05) different between *Nrf2^+/+^* and *Nrf2^-/-^* mice at 22 wk after urethane treatment are clustered into 4 expression profile groups (set 0–3). Expression level of each gene was normalized to that of *Nrf2^+/+^* saline controls and indicated as relative log ratio [log_2_ (normalized average intensity)]. Genes are listed in Dataset 2 and in Table 2. Sal = saline-treated lung, UN = uninvolved tissue, Tum = tumor tissue. (D) Among the Nrf2-dependently changed genes in pre-/early neoplastic stage at 12 wk (n = 118) and in tumors at 22 wk (n = 376), Venn diagram analysis identified gene transcripts that were changed at either time point (12 and 22 wk) and/or were genes in common between the two time points (i.e. 12 genes).

Because we found Nrf2-dependent differences in tumorigenesis, we used trancriptomic profiling of tumors and uninvolved tissues to identify gene networks that provide potential mechanistic insight to Nrf2-mediated effects. Gene transcripts (n = 118) that were significantly different between *Nrf2^+/+^* and *Nrf2^-/-^* mice 12 wk after urethane treatment (Table 1; n = 3 mice/group) were categorized primarily in cell-to-cell signaling and interaction, connective tissue development and function, cellular movement, tissue/cell morphology and cellular development, and inflammatory response and immune cell trafficking processes (Figure 6A; Figure S3A). Expression profiles were classified into 4 cluster sets (Figure 6B, Dataset S1). Transcripts highly up-regulated by urethane in *Nrf2^-/-^* mice relative to *Nrf2^+/+^* mice (set 0) included melanoma antigen (*Mela*), growth arrest specific 6 (*Gas6*), integrin alpha 6 (*Itga6*), matrix metalloproteinase 2 (*Mmp2*), CD34 antigen (*Cd34*), D site albumin promoter binding protein (*Dbp*), and transforming growth factor, beta induced (*Tgfbi*). Other gene clusters (sets 1–3) that were relatively suppressed in *Nrf2^-/-^* mice compared to *Nrf2^+/+^* mice (Figure 6B, Table 1, Dataset S1) included genes for ARE-responsive antioxidants, solute carrier family, chemokines, arginase type II (*Arg2*), and attractin like 1 (*Atrnl1*).

**Table 1:** Representative lung genes from a total of 118 that were significantly (p<0.05) different between *Nrf2^+/+^* and *Nrf2^-/-^* mice 12 wk after beginning urethane treatment.

GI Accession	Gene Symbol	Gene Description	Major Gene Ontology (GO) Categories	Cluster (Figure 7A)
**Cell-To-Cell Signaling and Interaction, Connective Tissue Development and Function**				
NM_019521	*Gas6*	growth arrest specific 6	regulation of cell growth, calcium ion binding	0
NM_009369	*Tgfbi*	transforming growth factor, beta induced	cell adhesion	0
NM_008610	*Mmp2*	matrix metallopeptidase 2	blood vessel maturation, collagen catabolic process, peptidoglycan metabolic process	0
NM_133654	*Cd34* *	CD34 antigen	cell adhesion, leukocyte migration	0
BM935811	*Itga6*	integrin alpha 6	cell adhesion, leukocyte migration	0
AI987929	*Ndrg1*	N-myc downstream regulated gene 1	cell differentiation, mast cell activation	1
BF168458	*Timp2*	tissue inhibitor of metalloproteinase 2	negative regulation of cell proliferation, regulation of MAPKKK cascade	3
**Glutathione metabolism, Nrf2-mediated oxidative Stress Response, Drug metabolism**				
NM_011034	*Prdx1* *	peroxiredoxin 1	regulation of NF-kappa B import into nucleus, response to oxidative stress	1
NM_010497	*Idh1*	isocitrate dehydrogenase 1 (NADP+), soluble	glutathione metabolic process, response to oxidative stress, glyoxylate cycle,	1
NM_015762	*Txnrd1* *	thioredoxin reductase 1	cell proliferation, cell redox homeostasis	2
NM_009676	*Aox1* *	aldehyde oxidase 1	electron transport	2
BC019434	*Ugt1a2* *	UDP glucuronosyltransferase 1 family, polypeptide A2	transferase activity	2
NM_008180	*Gss*	glutathione synthetase	glutathione biosynthetic process	2
BM210600	*Srxn1*	sulfiredoxin 1 homolog	response to oxidative stress	3
NM_009656	*Aldh2*	aldehyde dehydrogenase 2	oxidation reduction, metabolic process	3
NM_181415	*Atrnl1* *	attractin like 1	G-protein coupled receptor protein signaling	3
**Organ Development, Gene expression, Cancer**				
BI247018	*Ptpmt1*	protein tyrosine phosphatase, mitochondrial 1	phosphatidylinositol metabolic process, protein amino acid dephosphorylation	0
AW493043	*Zfp346*	zinc finger protein 346	apoptosis, double-stranded RNA binding	0
BG074964	*Sra1*	steroid receptor RNA activator 1	apoptosis, cell differentiation and proliferation	1
AF061972	*Htatip2*	HIV-1 tat interactive protein 2	angiogenesis, apoptosis, cell cycle	2
NM_016974	*Dbp*	D site albumin promoter binding protein	regulation of transcription, DNA-dependent, regulation of cell proliferation	0
**Immune responses**				
NM_020001	*Clec4n*	C-type lectin domain family 4, member n	proteolysis, macromolecule catabolic process, immune response	2
NM_011355	*Sfpi1*	hematopoietic transcription factor PU.1, SFFV proviral integration 1	granulocyte, lymphocyte, and macrophage differentiation	2
AF128196	*Ccl9*	chemokine (C-C motif) ligand 9	chemotaxis, immune response	3
NM_009140	*Cxcl2*	chemokine (C-X-C motif) ligand 2	chemotaxis, immune response	3
**Cellular Compromise, Cellular Growth and Proliferation**				
BB188812	*Ptpn5*	protein tyrosine phosphatase, non-receptor type 5	protein amino acid dephosphorylation, phosphoric monoester hydrolase activity	0
AK016670	*Bcl2l14*	BCL2-like 14	Apoptosis facilitator, regulation of cell death	1
**Lipid catabolism**				
AK004768	*Osbpl3*	oxysterol binding protein-like 3	lipid transport, steroid metabolic process	0
BI111416	*Echs1*	enoyl Coenzyme A hydratase, short chain, 1, mitochondrial	fatty acid metabolic process, lipid metabolic process	1
AU067703	*Sc5d*	sterol-C5-desaturase homolog	lipid biosynthetic process, metabolic process	3
**Molecular Transport**				
AK010399	*Snx10*	sorting nexin 10	cell communication, protein transport	1
AV371434	*Slc5a1*	solute carrier family 5, member 1	sodium/glucose cotransporter	2
NM_013612	*Slc11a1*	solute carrier family 11, member 1	proton-coupled divalent metal ion transporters	3
**Others**				
BB794642	*Mela*	melanoma antigen	RNA-dependent DNA replication, proteolysis, DNA integration, viral infectious cycle	0
NM_009705	Arg2	arginase type II	arginine metabolic process, negative regulation of nitric-oxide synthase activity	1

*Genes significantly different in lung tumors between *Nrf2^+/+^* and *Nrf2^-/-^* mice at 22 wk.

#### Nrf2-dependent transcriptomics in tumors (22 wk)

Urethane caused Nrf2-dependent changes in a small number of the gene transcripts (n = 11) in the tumor microenvironment (UN) at 22 wk relative to controls. Transcripts included polymerase, delta (*Poldip2*), sonic hedgehog (*Shh*), and ribonuclease T2 (*Rnaset2*). IPA indicated that tumor microenvironment participates in a network of cellular growth and proliferation, tumor morphology, and cellular development (Figure S3B; n = 3 mice/group). A number of genes (n = 376) were significantly changed in tumor tissues relative to vehicle-treated tissues in a Nrf2-dependent manner at 22 wk (Table 2, Dataset S2). The largest family of genes encodes thiol metabolism enzymes (e.g. glutathione-S-transferase family), solute carrier family members, and transmembrane proteins. Predominant functional categories of Nrf2-dependently changed tumor genes at 22 wk (Figure 6A, bottom panel) were largely similar to those of Nrf2-dependent lung genes determined at the 12 wk early neoplastic stage (Figure 6A, top panel). However, Nrf2 effector genes in tumor tissue were more closely associated with drug metabolism, cell cycle and death and organismal survival, and tumor morphology (Figure 6A, Figure S3C). Canonical pathway analysis by IPA determined that Nrf2 effector genes in lung tumors overlaid multiple signal transduction mechanisms including predicted glutathione metabolism (12 out of 98 pathway molecules match, 12/98), oxidative stress response (14/183), aryl hydrocarbon receptor signaling (12/154), LPS/IL-1 mediated inhibition of RXR function (14/213), glioblastoma multiforme signaling (12/163), and others (Dataset S3). Expression profiles of these 376 Nrf2-dependent tumor transcripts were classified (Figure 6C, Dataset S2); subsets of tumor genes that were relatively less-responsive to urethane (set 0) or more suppressed (set 1) in *Nrf2^-/-^* than in *Nrf2^+/+^* mice involved cell cycle, antioxidant regulation, cell-cell interaction, and metabolism. Some antioxidants and solute carrier family genes were differentially down-regulated in tumors (set 2), while vasculature, immunity, and other antioxidant genes were differentially up-regulated in tumors (set 3). Although major functional categories of Nrf2-dependent transcripts were similar in early neoplastic lungs and in tumors (see Figure 6B), only 21 individual genes were found to be common at these two stages (Figure 6D). Among them, *Cd34*, protein tyrosine phosphatase, receptor type, F (*Ptprf*), *Txnrd1*, *Pgd, Ugt1*a1, *Gpr137b*, and dynein light chain Tctex-type 1 (*Dynlt1*) were connected in a molecular network of cancer-cell cycle-cell death (Figure S3C).

**Table 2:** Representative lung tumor gene transcripts from a total of 376 that were significantly (*p*<0.01).

GI Accession	Gene Symbol	Gene Description	Major Gene Ontology (GO) Categories	Cluster (Figure 7C)
**Cell cycle, Cell growth and proliferation, Cancer**				
NM_016904	*Cks1b*	CDC28 protein kinase 1b	cell cycle, cell division	0
NM_007631	*Ccnd1*	cyclin D1	re-entry into mitotic cell cycle, cell cycle	2
NM_013737	*Pla2g7*	phospholipase A2, group VII	inflammatory response, lipid catabolic process	0
NM_009367	*Tgfb2*	transforming growth factor, beta 2	angiogenesis, axon guidance, blood vessel development	0
NM_028618	*Dmkn*	dermokine	cell differentiation	3
NM_011176	*St14*	suppression of tumorigenicity 14	proteolysis, cell migration	2
NM_027950	*Osgin1*	oxidative stress induced growth inhibitor 1	cell differentiation, regulation of cell death	3
**Organismal injury and abnormality, Thiol and xenobiotic metabolism, Tumor morphology**				
NM_010358	*Gstm1*	glutathione S-transferase, mu 1	glutathione metabolic process	0
NM_013701	*Ugt1a1* *	UDPglucuronosyltransferase 1 family, polypeptide A1	transferase activity	3
NM_008012	*Akr1b8*	aldo-keto reductase family 1, member B8	oxidation reduction	1
NM_030677	*Gpx2*	glutathione peroxidase 2	response to oxidative stress	0
NM_181415	*Atrnl1* *	attractin like 1	G-protein coupled receptor protein signaling pathway	0
NM_027988	*Noxo1*	NADPH oxidase organizer 1	superoxide metabolic process, cell communication	0
NM_172442	*Dtx4*	deltex 4 homolog (Drosophila)	Notch signaling pathway	1
NM_010215	*Il4i1*	interleukin 4 induced 1	aromatic amino acid family metabolic process, regulation of Rab GTPase activity, oxidation reduction	0
**Cell-To-Cell Signaling and Interaction, Tissue Development, Cell morphology**				
NM_011923	*Angptl2*	angiopoietin-like 2	signal transduction	2
NM_008176	*Cxcl1*	chemokine (C-X-C motif) ligand 1	cellular process, immune response, inflammatory response	0
NM_010196	*Fga*	fibrinogen alpha chain	signal transduction, blood coagulation, platelet activation, protein polymerization	0
NM_010576	*Itga4*	integrin alpha 4	blood vessel remodeling, cell adhesion, cell migration	0
**Cardiovascular system development and function, Gene expression**				
NM_008124	*Gjb1*	gap junction membrane channel protein beta 1	cell communication, cell-cell signaling	0
NM_033601	*Bcl3*	B-cell leukemia/lymphoma 3	B cell and follicular dendritic cell differentiation	0
NM_027286	*Ace2*	angiotensin I converting enzyme 2	regulation of systemic arterial blood pressure by renin-angiotensin, proteolysis	3
NM_080457	*Muc4*	mucin 4	cell adhesion, cell-matrix adhesion	0
NM_009841	*Cd14*	CD14 antigen	immune response, inflammatory response	3
NM_009330	*Hnf1b*	HNF1 homeobox B	regulation of transcription, DNA-dependent	3
NM_010807	*Marcksl1*	MARCKS-like 1	positive regulation of cell proliferation	0
NM_011340	*Serpinf1*	serine (or cysteine) peptidase inhibitor, clade F, member 1	negative regulation of angiogenesis, positive regulation of neurogenesis	0
**Carbohydrate and lipid metabolism, Small molecule biochemistry**				
NM_019395	*Fbp1*	fructose bisphosphatase 1	glycolysis and gluconeogenesis	0
NM_008412	*Ivl*	involucrin	keratinocyte differentiation, keratinization,	0
NM_022411	*Slc13a2*	solute carrier family 13 member 2	sodium ion transport (sodium-dependent dicarboxylate transporter),	1
NM_019588	*Plce1*	phospholipase C, epsilon 1	activation of MAPK activity, lipid metabolic process	1
NM_25374	*Glo1*	glyoxalase 1	anti-apoptosis, carbohydrate metabolic process	0
NM_153133	Rdh9	retinol dehydrogenase 9	retinoid metabolic process, steroid metabolic process	0
NM_018737	*Ctps2*	cytidine 5'-triphosphate synthase 2	pyrimidine nucleotide biosynthetic process, glutamine metabolic process	0
**Others**				
NM_203320	*Cxcl3*	chemokine (C-X-C motif) ligand 3	chemotaxis, inflammatory response	3
NM_021564	*Fetub* *	fetuin beta	FXR/RXR activation signaling, regulation of the insulin and hepatocyte growth factor receptors	0

* Genes also significantly different between *Nrf2^+/+^* and *Nrf2^-/-^* mice at 12 wk after urethane treatment.

varied between *Nrf2^+/+^* and *Nrf2^-/-^* mice at 22 wk after urethane.

#### Validation of Nrf2-dependent transcripts identified at early and late stages of carcinogenesis

Differential mRNA expression of multiple ARE-responsive genes that were increased (aldo-keto reductase family 1, member B8, *Akr1b8*; aldehyde oxidase 3, *Aox3*; *Prdx1*) or decreased (aldehyde dehydrogenase 1 family, member A1, *Aldh1a1*; *Gstm;* glutathione peroxidase 2, *Gpx2*) in tumors were confirmed using qRT-PCR (Figure S4A). Total tissue glutathione (GSH) levels were also compared between the two genotypes to support Nrf2-dependent changes of many genes engaged in GSH synthesis mechanisms (e.g., glutathione synthetase, *Gss*; *Gclc*; glucose-6-phosphate dehydrogenase X-linked, *G6pdx*) after urethane treatment (Figure S4A). Non-antioxidant genes with Nrf2-dependent expression patterns were confirmed by qRT-PCR in 12 wk (*Mela*, *Cd34, Areg, Itga6, Arg2*, *Nek6*) and in 22 wk (*Cxcl1*, *Cks1b*, *Cdkn2c*, *Gjb1*,*Cd14*, prostaglandin I2 synthase, *Ptgis*), most of which were involved in the primary functional networks identified using the pathway analysis tool (Table 2, Figure S3).

## Discussion

We have found that murine Nrf2 has a cell survival role in lung cancer. That is, *Nrf2* deficiency reduced sensitivity to urethane-induced lung tumorigenesis. Importantly, *Nrf2* deficiency significantly enhanced lung inflammatory and cell injury responses during early pre-neoplastic stages. Transcriptomic analysis of the Nrf2-dependent gene transcript expression during early tumorigenesis (12 wk after urethane) identified specific pathways including cell-to-cell signaling and interaction, connective tissue development and function, glutathione metabolism and oxidative stress, and immune/inflammatory responses. Nrf2-dependent gene expression in the late stages of tumorigenesis was associated with cell cycle regulation, organismal injury/survival, xenobiotic/thiol metabolism, and cell-to-cell signaling. Many similarities were found in these functional categories between the early and late phases of tumorigenesis, while immunological responses early and cell cycle/growth and organismal survival in the more advanced stages were unique. Results suggest that reductions of ARE-responsive cytoprotective genes in tumor cells of *Nrf2* ^-/-^ mice render disadvantage to their survival during urethane-induced tumorigenesis and that other, non-antioxidant/defense pathways observed such as cell cycle and cell-to-cell signaling are essential components to this biological consequence. Together, the current findings support observations that activated Nrf2 is associated with an increased risk of human lung cancer [13].

Our results deviated from other experimental tumorigenesis models in non-pulmonary tissues (e.g., gallbladder, liver, stomach, colon, esophagus, skin, head/neck, prostate, bladder, mammary) where tumors or cancers were increased in *Nrf2^-/-^* mice relative to *Nrf2^+/+^* mice [19], [20], [21], [22], [23], [24], [25], [26], [27], [28]. However, a recent study found that experimentally implanted lung cancer cells (Lewis lung carcinomas) produced significantly more pulmonary metastatic nodules in *Nrf2* ^-/-^ than in *Nrf2^+/+^*mice, while the metastatic nodule formation was suppressed in *Keap1*-knockdown mice compared to *Keap1* normal mice [29]. The authors suggested that Nrf2 in host lung tissues prevents pulmonary metastasis through the stabilization of the redox balance in the hematopoietic and immune cells by increasing immunosuppressive cells which can lead to a decrease in CD8^+^ T-cell immunity. We also tested the role of Nrf2 in a 2-stage tumorigenesis model using 3-methylcholanthrene (3-MCA) as the initiator followed by butylated hydroxytoluene (BHT) as the promoter. Interestingly, no differences in tumor multiplicity were found between *Nrf2^-/-^* and *Nrf2^+/+^* mice (data not shown), which suggested that the molecular pathways required for urethane-induced tumorigenesis are different from the 2-stage model. Given the potential role for Nrf2 in protection from cytotoxicity during the urethane-induced tumor initiation stage, it is possible that the cell survival or beneficial nature of Nrf2 may depend on chemical carcinogens and on the stages of carcinogenesis (i.e., initiation, promotion, or metastasis) in mice. Therefore, assessment of other models, specifically those bypassing inflammation and injury phase during initiation or those focused on the advanced stages, well past tumor promotion, may provide additional insight to the characteristics of pulmonary Nrf2.

Promotion of cell survival is a phenotype that has been linked to Nrf2. In embryonic fibroblasts and HepG2 cells, cell survival involves adaptation to deal with the increased ROS called "an adaptive survival response" and in some cases involves an Nrf2 effector, heme oxygenase-1 [30]. In the current study we found that not only well-known ARE responsive genes (e.g., *Prdx1*, *Ugt*) but other novel Nrf2-dependent redox genes such as *Akr1b8*, NADPH oxidase organizer 1 (*Noxo1*), and solute carrier genes (e.g., *Slc13a2*) may have a role in cellular survival during lung tumorigenesis. Recent studies determined that permanent or constitutive NRF2 activation in human primary lung cancer cells and lung cancer cell lines (e.g., A549, H460) with *KEAP1* and/or *NRF2* somatic mutations was associated with enhanced chemoresistance and radioresistance, and siRNA inhibition of *NRF2* restored the chemosensitivity of these cells [15], [25], [28], [31]. Chemoresistance observed in these studies resulted from the overexpression of ARE-responsive genes encoding drug efflux pumps including multidrug resistant–associated protein (*Mrp*). This indicates that permanent NRF2 activation may be a novel biomarker in lung cancers and that Nrf2 inhibitors may provide increased efficacy for the treatment of cancers in specific patients with abnormally high NRF2-ARE activation.

The current study also further supports a role of inflammation in the urethane-induced carcinogenesis model [32], and suggests the association of multiple inflammatory mediators, particularly *Ptgis* and *Cxcl1*, with Nrf2-dependent tumorigenesis. PTGIS, the enzyme that synthesizes prostacyclin, is anti-inflammatory in the lung [33]. Importantly, a 92% reduction in tumor multiplicity was found in *Ptgis*-overexpressing mice [33] and prostacyclin analogs are currently in use for human NSCLC clinical trials in the National Cancer Institute Lung Cancer Biomarker and Chemoprevention Consortium. Heightened *Ptgis* expression in tumors of *Nrf2* ^-/-^ mice suggested that tumor microenvironment suppressed the normal inflammatory response in these mice. We also found persistent neutrophilic infiltration and concurrent induction of *Cxcl1* (a neutrophil chemoattractant) in *Nrf2* ^+/+^ mice by 22 wk, but not in *Nrf2* ^-/-^ mice. Neutrophils are often observed during the more advanced stages of lung tumor development, specifically urethane-induced [32], and are thought to be involved in the production of ROS as indicated by skin carcinogenesis models [34].

Our transcriptome analysis also identified numerous urethane-dependent genes in lung tumors of wild-type mice (BALB/cCR), and the functional categories of these neoplastic genes (e.g., genetic disorder, cardiovascular system function, cell death, immunological disease) were largely not concordant with those of genes changed at the early-neoplastic stage (e.g., small molecular biochemistry, nucleic acid metabolism, cell morphology, lipid metabolism, cell cycle, molecular transport, protein trafficking) and in uninvolved tissues of tumor-bearing lungs (e.g., small molecular biochemistry, immune cell trafficking, hematopoiesis, nucleic acid metabolism, drug metabolism, nerve system function). These findings indicate distinct biochemical and molecular changes are occurring in tumor tissues, while transcriptomics in the pre/early neoplastic stage and in uninvolved tumor microenvironments are in general similar. However, transcriptome changes in cellular growth and proliferation-associated genes were common throughout all tissues and neoplastic stages. Genes such as claudin 3 (*Cldn3*), thymidylate synthetase (*Tyms*), and thymidine kinase 1 (*Tk1*) in these tumors were previously found in a single injection urethane model (24–42 wk following urethane) in tumors of A/J mice [35]. In addition, comparison with toll-like receptor 4 (*Tlr4*) dependent genes in a 2-stage (MCA/BHT) mouse pulmonary carcinogenesis model [36] identified many genes (e.g. angiotensin I converting enzyme 2, *Ace2*; Bcl2-like 14,*Bcl2l14*; claudin 2, *Cldn2*) also to be Nrf2-dependent. Genes changed by urethane in the early neoplastic stage that also differed in the MCA-BHT model [36] included amphiregulin (*Areg*), arginase 1 (*Arg1*), chemokine receptor 2 (*Ccr2),* cyclin-dependent kinase inhibitor 1A (*Cdkn1a*), epiregulin (*Ereg*), gap junction protein, alpha 1 (*Gja1*), and secreted phosphoprotein 1 (*Spp1*). Overall from previous and current tumor analyses using different dosing regimens, carcinogens, and strains of mice, a panel of common transcripts (e.g. *Arg1*, *Areg*, *Ereg*, *Ccr2*, *Cdkn1a*, *Gja1*, *Ptgis*, *Spp1*) may provide a useful tool for early diagnosis of lung neoplasm.

**Figure 7:**
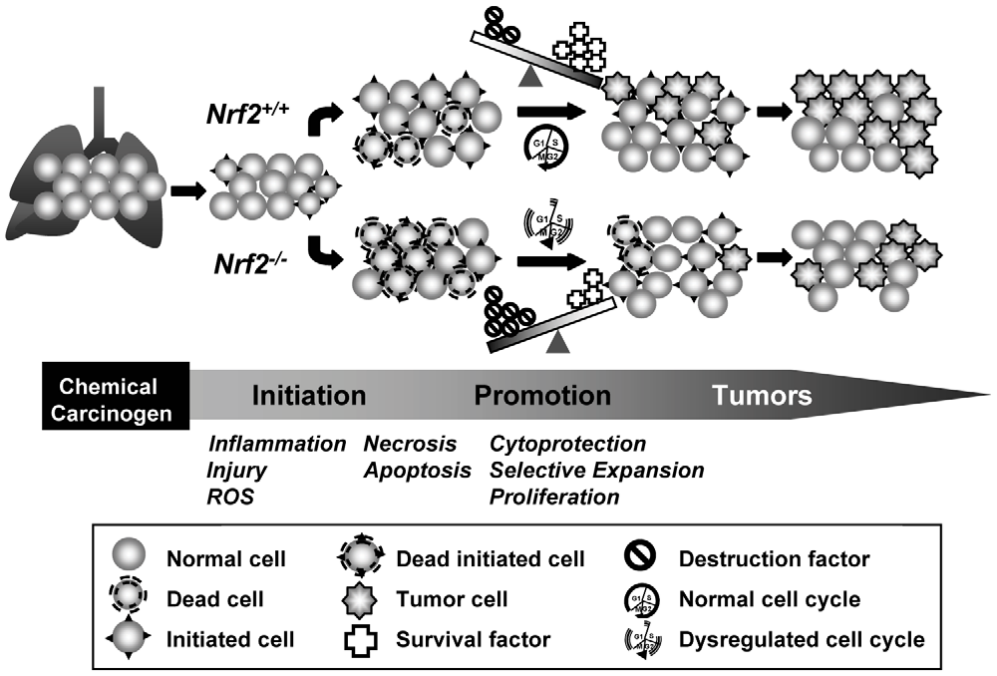
Proposed role for Nrf2 in urethane-induced lung tumorigenesis. Urethane treatment causes pulmonary inflammation and injury during the pre-neoplastic stage, which results in overproduction of reactive oxygen species (ROS) and cellular death by necrosis and apoptosis. Compared to *Nrf2* ^+/+^ mice, *Nrf2^-/-^* mice have lowered cell survival factors including cellular redox and drug metabolism enzymes (e.g., glutathione synthetase, UDP glucuronosyl transferase 1 family) and cell maintenance systems including numerous metabolic enzymes and transport proteins (e.g., solute carrier family). These mice therefore have heightened cellular destruction factors (e.g., ROS, airway secretion, inflammation), which overwhelms cellular cytoprotection tools and causes mass death of injured cells including tumor initiated cells during the pre-neoplastic stage. *Nrf2^-/-^* mice also exhibit dysregulated expressions of many genes involved in cell cycle and death (e.g., CDC28 protein kinases, cyclin D1, cyclin dependent kinase inhibitor 2C, B-cell leukemia/lymphoma 6, unc-119 homolog) relative to wild type mice during tumorigenesis. Overall, increased susceptibility to acute injury due to lack of survival signals leading to net cell loss is beneficial to *Nrf2^-/-^* mice for their tumor suppression.

In conclusion, our novel results provide evidence that Nrf2 contributes to lung tumorigenesis susceptibility. We propose that during pulmonary inflammation and injury in the early stages of tumor development induced by urethane, lung cells in *Nrf2* ^-/-^ mice had reduced cell survival factors such as cellular redox and drug metabolism enzymes and cell maintenance system, relative to those in *Nrf2^+/+^* cells (see hypothetical schematic, Figure 7). Consequently, *Nrf2* ^-/-^ mice had decreased cytoprotection and massive death of initiated cells that would normally develop into adenomas (Figure 7). Nrf2-dependent genes associated in cell cycle and cell death also affect differential proliferation of these initiated tumor progenitor cells between *Nrf2^+/+^* and *Nrf2^-/-^* mice (Figure 7). Overall, increased susceptibility to urethane-induced pre-neoplastic injury leading to net cell loss due to lack of cell survival signals in *Nrf2* ^-/-^ mice is paradoxically beneficial to their tumor suppression. Results indicate the importance of understanding the pre-neoplastic pathologic events as well as understanding the mechanisms through which cancer chemotherapy using supplemental antioxidants modulates multi-stage carcinogenesis. In addition to this unique notion, our transcriptomal analysis provides new insights into the downstream molecular mechanisms of Nrf2 in a pulmonary neoplastic microenvironment.

## Materials and Methods

### Ethics Statement

All animal use was approved by the National Institute of Environmental Health Sciences Animal Care and Use Committee under protocol number P03-05 and follows the Helsinki convention for the use and care of animals.

#### Animals and carcinogen treatment

Female wild-type mice (BALB/cCR(Japan)-*Nrf2^+/+^)* and *Nrf2* deficient (BALB/cCR(Japan)-*Nrf2^-/-^*) littermates were generated from a breeding colony at the National Institute of Environmental Health Sciences (NIEHS) animal facility. Animals were housed in a virus- and antigen-free room. Water and mouse chow (modified AIN-76A, Harlan Teklad, Madison, WI) were provided *ad libitum*. Mice (4–6 wk) were injected with urethane (1 mg per *g* BW in saline; Sigma, St. Louis, MO) or with vehicle (saline) i.p. weekly for 7 wk. Mice were sacrificed at 9, 11, 12, or 22 wk after the first injection by Nembutal overdose (104 g/Kg BW).

#### Lung fixation, tumor analysis, histopathology and immunohistopathology

Lung tissues were fixed with Tellyesniczky's fixative (22 wk post injection) or 10% neutral buffered formalin (other times) for histopathology and tumor analysis as described previously [37]. Apoptotic cell death was determined on lung tissue sections *in situ* using a TUNEL kit (Promega, Madison, WI) as described previously [38]. See Methods S1 for additional details.

#### BAL cell analyses and myeloperoxidase assay

The right lung of each mouse was lavaged *in situ*, four consecutive times with HBSS (0.5 ml/25 g BW). BAL fluid was then analyzed for total protein content (a marker of lung permeability change) and numbers of epithelial (a marker of epithelial cell injury) and inflammatory cells following published procedures using a modified Wright’s stain (Diff-Quik; Baxter Scientific Products, McGaw Park, IL) [36]. Due to cellular lysis in BAL which prohibited neutrophil enumeration, neutrophil abundance in BAL was quantified through measurement of neutrophil myeloperoxidase (MPO) using a colorimetric analysis kit (Cytostore, Canada) which assessed conversion of hydrogen peroxide to oxygen radicals by MPO. Cell viability and cytotoxicity were determined by the amount of released LDH in BAL aliquots (50 µl) using a colorimetric assay (Sigma-Aldrich, St. Louis, MO).

#### Tissue protein isolation

Tumors (involved regions) were micro-dissected from lungs and remaining uninvolved tissue (UN) was used as a control in urethane-treated lungs at 22 wk. Snap frozen tumor, UN, or whole lungs (saline-treated controls and urethane-treated lungs at 12 wk) were homogenized (n = 3/group) in RIPA buffer. Nuclear proteins were isolated from pulverized lung samples following procedures described elsewhere [39]. Proteins were quantified and stored in aliquots at −70°C.

#### Electrophoretic mobility shift assay (EMSA)

Nuclear DNA binding activity of Nrf2 was determined by EMSA analyses of nuclear protein aliquots (5 µg) on 3×10^4^ cpm [γ^32^P] ATP end-labeled ARE consensus sequence following procedures described previously [40]. Specific binding activity for Nrf2 and small Maf protein was determined by pre-incubation of nuclear proteins with anti-Nrf2 (sc-722x, Santa Cruz Biotechnology, Santa Cruz, CA) and anti-small Maf (F/K/G) antibody (sc-22831x, Santa Cruz ), respectively, followed by EMSA.

#### Immunoblot analyses and GSH measurement

Lung total (30–50 µg) or nuclear (10 µg) proteins were separated on appropriate percentage Tris-HCl SDS-PAGE gels (Bio-Rad, Hercules, CA) and analyzed by routine immunoblotting using specific antibodies against Ki67 (Abcam Inc., Cambridge, MA), Nrf2 (Santa Cruz), lamin B1 (sc-20682, Santa Cruz), or actin (sc-1615, Santa Cruz). Representative protein blot images from multiple analyses were scanned by a Bio-Rad Gel Doc system (Hercules, CA). Total GSH level was determined in lung RIPA homogenates by a colorimetric method following manufacturer instructions (Northwest Life Science Specialties, LLC, Vancouver, WA).

#### Affymetrix cDNA Microarray Analysis

Total RNA was isolated from homogenates of total lung, UN or TU tissues (n = 3/group) using Qiagen RNeasy Mini kits (Qiagen Inc., Valencia, CA). Microarray processing was done by the NIEHS Microarray Core Facility as described previously [36] with Affymetrix mouse genome 430_2.0 microarrays using Affymetrix reagents and protocols (Santa Clara, CA). GeneSpring Expression Analysis (Agilent Technologies, Inc., Santa Clara, CA) was used for statistical analyses and characterization of data and Ingenuity Pathway Analysis (IPA) software (www.ingenuity.com, Ingenuity Systems, Inc., Redwood City, CA) identified potentially significant functional connections and mechanistic pathways. Characterization of the microarray data is described in Methods S1.

#### Quantitative reverse transcriptase-polymerase chain reaction (qRT-PCR)

An aliquot (1 µg) of total lung RNA was reverse transcribed as previously published [40] and gene specific primers used in quantitative RT-PCR reactions, similar to previous publications [33]. For additional details, see Methods S1.

#### Statistical analysis of non-microarray data

Data were expressed as group mean±standard deviation. Two-way ANOVA was used to evaluate effects of treatment (vehicle, urethane) between two genotypes (*Nrf2^+/+^*, *Nrf2^-/-^*). The Student-Newman-Keuls test was used for *a posteriori* comparisons of means (*p*<0.05). All statistical analyses were performed using the SigmaStat 3.0 software program (SPSS Science Inc., Chicago, IL).

## Supporting Information

Figure S1 **Airway cytotoxicity and secretion in pre-neoplastic stage.** Giemsa-stained cytocentrifuge slides of bronchoalveolar lavage (BAL) fluid show increased BAL cell populations and greater cell clustering and lysis caused by urethane in *Nrf2^-/-^* than in *Nrf2^+/+^* mice before tumor development at 9 wk (inset: higher magnification of BAL cells). Enhanced airway secretion (black arrow heads) and epithelial mucous production (black arrows) as indicated by AB/PAS-stained bronchial airway sections at 9 wk was consistent with clustering of the BAL cells in *Nrf2^-/-^* mice (inset: lower magnification of proximal airway and airspace). At 11 wk, clustering was resolved but greater cell lysis and cell debris in *Nrf2^-/-^* mice were accompanied by appearance of macrophages with phagocytosis-like features (red arrows) Insets present higher magnification of phagocytic macrophages. Representative slides showing intermediate magnitude of pathology for each treatment group are presented. AV, alveoli; BR, bronchi; PA, pulmonary artery. Bars indicate 100 µm.
(TIF)

Figure S2 **Top functional networks of significantly changed gene transcripts in lung tumors of** ***Nrf2^+/+^*** **mice at 22 wk.** Ingenuity Pathway Analysis (IPA) generated essential functional networks of the genes significantly (≥2-fold, n = 3461) changed in *Nrf2^+/+^* tumors. Highest association score (40) was for the genetic disorder-skeletal and muscular disorders- developmental disorder network (A) where the genes such as coiled-coil domain containing 85A, (*Ccdc85a*), dystrophin, muscular dystrophy (*Dmd*), and fyn proto-oncogene (*Fyn*) were mapped. Many genes (e.g., CCAAT/enhancer binding protein (C/EBP) alpha, *Cebpa*; lymphotoxin B, *Ltb*) were identified for cellular development-cellular growth and proliferation-hematological system development and function-hematopoiesis networks (B and C, Scores 34 and 33, respectively) and others related in cell cycle- cellular movement-cancer network (D, score 33, e.g., p21, *Cdkn1a*; cell division cycle 20, *Cdc20*) or in amino acid metabolism-molecular transport-small molecular biochemistry (E, Score 33, e.g., platelet derived growth factor receptor, beta polypeptide *Pdgfrb*; platelet-activating factor acetylhydrolase, isoform 1b, subunit 3, *Pafah1b3*) were also closely associated during tumorigenesis. (.ppt).
(PPT)

Figure S3 **Top functional networks of Nrf2-dependent genes changed during urethane-induced tumorigenesis.** (A) Ingenuity Pathway Analysis (IPA) generated a key network (Score 54) of cell-to-cell signaling and interaction-connective tissue development and function-ophthalmic disease with Nrf2-dependently regulated genes in pre-/early-neoplastic microenvironment (12 wk), in which genes encoding matrix metalloproteinase 2 (*Mmp2*) and D site albumin promoter binding protein (*Dbp*) were mapped as core molecules. (B) Nrf2-dependent lung tumor genes were highly associated in the networks of cell cycle-cancer-connective tissue development and function (Score 43, e.g., cyclin D1, *Ccnd1*; E2F transcription factor 3, *E2f3*), cell-to-cell signaling and interaction-tissue development-cell function (Score 38, e.g., chemokine (C-X-C motif) ligand 1, *Cxcl1*; integrin alpha 4, *Itga4*), and tumor morphology-cancer-dermatological disease and conditions (Score 38, e.g., G protein-coupled 56, *Gpr56*; glutathione-S-transferase, alpha 3, *Gsta3*) as generated by IPA. (C) Nrf2-dependently modulated genes in common (n = 21 genes) in early-neoplastic microenvironment (12 wk) and in lung tumors (22 wk) were functionally associated in cancer-cell cycle-cell death network (Score 23). They included genes encoding CD34, UGT1a1, fetuin beta (*Fetub*), G protein-coupled receptor 137B (*Gpr137b*), ATP binding cassette, subfamily C, member 4 (*Abcc4*), phosphogluconate dehydrogenase (*Pgd*), etc. (.ppt).
(PPT)

Figure S4 **Confirmation of microarray transcript profiles.** (A) Expression of selected antioxidant/defense gene transcripts that were found by microarray analysis to be significantly increased or decreased by urethane in tumor tissues (22 wk): aldo-keto reductase family 1, member B8 (*Akr1b8*), aldehyde oxidase 3 (*Aox3*), peroxiredoxin 1 (*Prdx1*), glutathione peroxidase 2 (*Gpx2*), glutathione-S-transferase-µ (*Gstm*), and aldehyde dehydrogenase 1 family, member A1 (*Aldh1a1*). Relative suppression of these cytoprotective genes was evident in *Nrf2^-/-^* mice. Total glutathione (GSH) was determined in whole lung homogenates using a colorimetric kinetic analysis to support the differential induction of key GSH synthesis enzymes (e.g., glutathione synthetase, *Gss*; glutamate-cystein ligase, catalytic subunit, *Gclc*; glucose-6-phosphate dehydrogenase X-linked, *G6pdx*) between *Nrf2^+/+^* and *Nrf2^-/-^* mice during the tumorigenesis. Mean±SEM are presented (n = 3/group). *, p<0.05 vs. genotype-matched saline control mice. +, p<0.05 vs. treatment-matched *Nrf2^+/+^* mice. Expression of selected lung genes significantly varied between *Nrf2^+/+^* and *Nrf2^-/-^* mice at 12 wk (B) and at 22 wk (C) were determined by qRT-PCR. All qRT-PCR graphs depicted fold differences relative to *Nrf2^+/+^* saline level of 18s-normalized data. All qRT-PCR data are represented as group mean±SEM (n = 3/group).
(TIFF)

Table S1 **Representative lung genes significantly changed by urethane at 12 wk in** ***Nrf2^+/+^*** **mice (n = 6519,** ***p*** **<0.05).**
(DOC)

Table S2 **Top functional networks and involved genes significantly (** ***p*** **<0.05) changed in uninvolved tissues and in tumors of** ***Nrf2^+/+^*** **mice at 22 wk.**
(DOC)

Dataset S1 **12 wk Saline vs. Urethane Nrf2-WT vs. Nrf2-KO.**
(XLS)

Dataset S2 **Genes differentially changed between Nrf2-WT and Nrf2-KO mice by urethane in tumors at 22 wk (n = 376).**
(XLS)

Dataset S3 **Canonical pathways for 376 tumor genes significantly varied between Nrf2+/+ and Nrf2-/- mice at 22 wk.**
(XLS)

Methods S1 (DOC)
