# Supplementary figures and images for: Targeted Deletion of Nrf2 Reduces Urethane-Induced Lung Tumor Development in Mice
Source: PLoS One. 2011 Oct 21;6(10):e26590. doi: 10.1371/journal.pone.0026590 (PMC3198791; doi:10.1371/journal.pone.0026590)

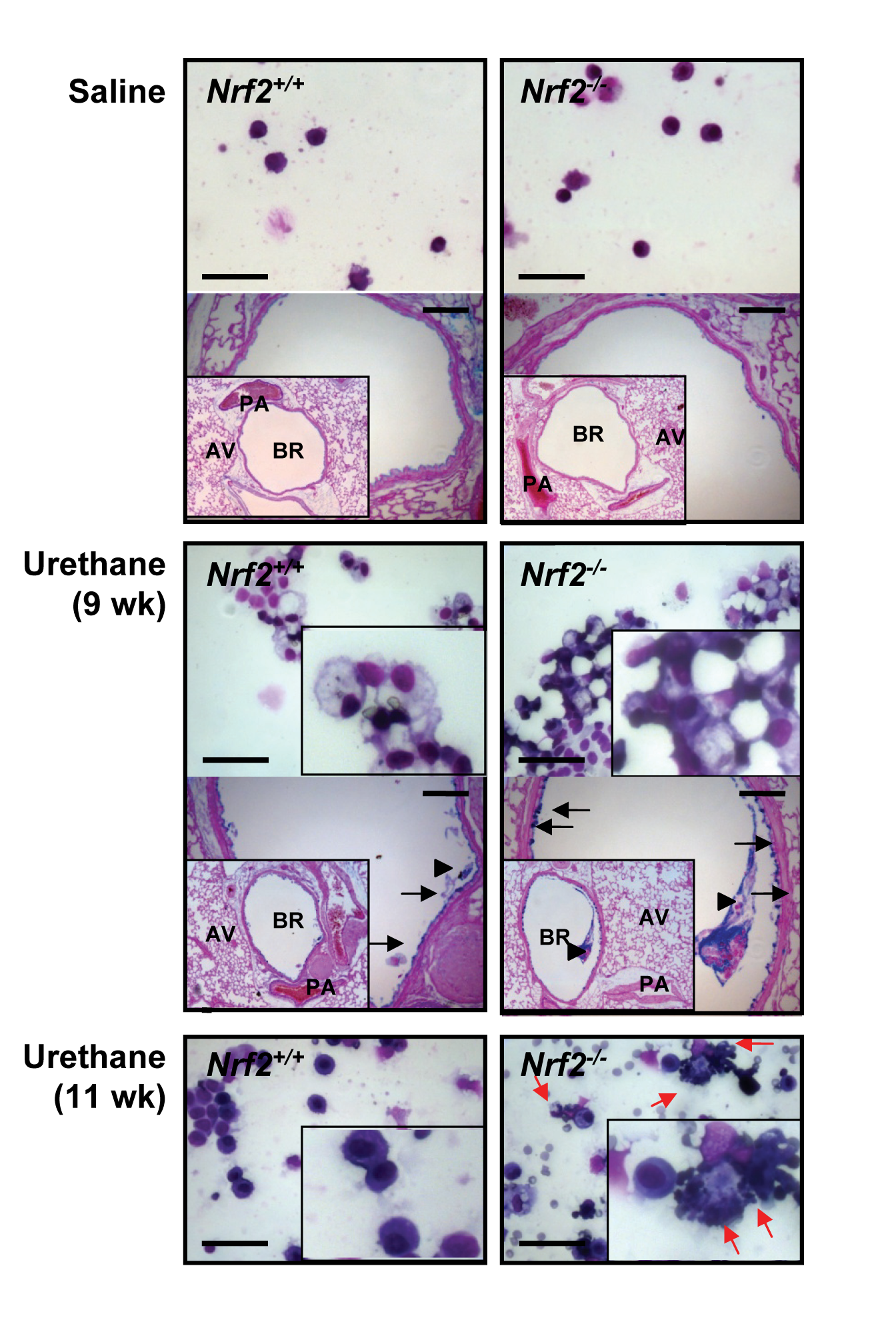

Supplement: Figure S1 — Airway cytotoxicity and secretion in pre-neoplastic stage. Giemsa-stained cytocentrifuge slides of bronchoalveolar lavage (BAL) fluid show increased BAL cell populations and greater cell clustering and lysis caused by urethane in Nrf2-/- than in Nrf2+/+ mice before tumor development at 9 wk (inset: higher magnification of BAL cells). Enhanced airway secretion (black arrow heads) and epithelial mucous production (black arrows) as indicated by AB/PAS-stained bronchial airway sections at 9 wk was consistent with clustering of the BAL cells in Nrf2-/- mice (inset: lower magnification of proximal airway and airspace). At 11 wk, clustering was resolved but greater cell lysis and cell debris in Nrf2-/- mice were accompanied by appearance of macrophages with phagocytosis-like features (red arrows) Insets present higher magnification of phagocytic macrophages. Representative slides showing intermediate magnitude of pathology for each treatment group are presented. AV, alveoli; BR, bronchi; PA, pulmonary artery. Bars indicate 100 µm. (TIF) [file pone.0026590.s001.tif]

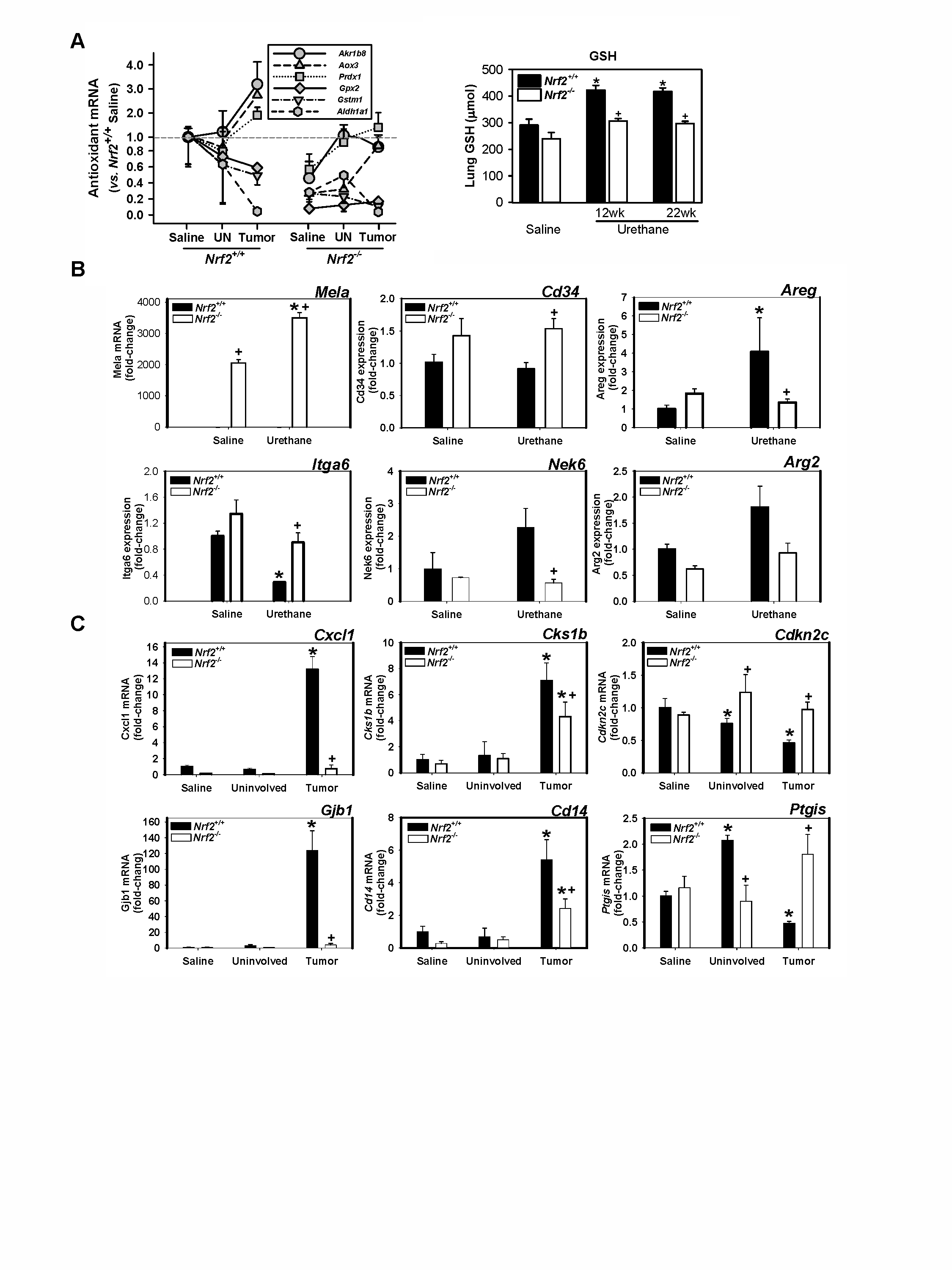

Supplement: Figure S4 — Confirmation of microarray transcript profiles. (A) Expression of selected antioxidant/defense gene transcripts that were found by microarray analysis to be significantly increased or decreased by urethane in tumor tissues (22 wk): aldo-keto reductase family 1, member B8 (Akr1b8), aldehyde oxidase 3 (Aox3), peroxiredoxin 1 (Prdx1), glutathione peroxidase 2 (Gpx2), glutathione-S-transferase-µ (Gstm), and aldehyde dehydrogenase 1 family, member A1 (Aldh1a1). Relative suppression of these cytoprotective genes was evident in Nrf2-/- mice. Total glutathione (GSH) was determined in whole lung homogenates using a colorimetric kinetic analysis to support the differential induction of key GSH synthesis enzymes (e.g., glutathione synthetase, Gss; glutamate-cystein ligase, catalytic subunit, Gclc; glucose-6-phosphate dehydrogenase X-linked, G6pdx) between Nrf2+/+ and Nrf2-/- mice during the tumorigenesis. Mean±SEM are presented (n = 3/group). *, p<0.05 vs. genotype-matched saline control mice. +, p<0.05 vs. treatment-matched Nrf2+/+ mice. Expression of selected lung genes significantly varied between Nrf2+/+ and Nrf2-/- mice at 12 wk (B) and at 22 wk (C) were determined by qRT-PCR. All qRT-PCR graphs depicted fold differences relative to Nrf2+/+ saline level of 18s-normalized data. All qRT-PCR data are represented as group mean±SEM (n = 3/group). (TIFF) [file pone.0026590.s004.tiff]
