# Supplementary material for: Targeted Deletion of Nrf2 Reduces Urethane-Induced Lung Tumor Development in Mice
Source: PLoS One. 2011 Oct 21;6(10):e26590. doi: 10.1371/journal.pone.0026590 (PMC3198791; doi:10.1371/journal.pone.0026590)
Supplement: Figure S2 — Top functional networks of significantly changed gene transcripts in lung tumors of Nrf2+/+ mice at 22 wk. Ingenuity Pathway Analysis (IPA) generated essential functional networks of the genes significantly (≥2-fold, n = 3461) changed in Nrf2+/+ tumors. Highest association score (40) was for the genetic disorder-skeletal and muscular disorders- developmental disorder network (A) where the genes such as coiled-coil domain containing 85A, (Ccdc85a), dystrophin, muscular dystrophy (Dmd), and fyn proto-oncogene (Fyn) were mapped. Many genes (e.g., CCAAT/enhancer binding protein (C/EBP) alpha, Cebpa; lymphotoxin B, Ltb) were identified for cellular development-cellular growth and proliferation-hematological system development and function-hematopoiesis networks (B and C, Scores 34 and 33, respectively) and others related in cell cycle- cellular movement-cancer network (D, score 33, e.g., p21, Cdkn1a; cell division cycle 20, Cdc20) or in amino acid metabolism-molecular transport-small molecular biochemistry (E, Score 33, e.g., platelet derived growth factor receptor, beta polypeptide Pdgfrb; platelet-activating factor acetylhydrolase, isoform 1b, subunit 3, Pafah1b3) were also closely associated during tumorigenesis. (.ppt). (PPT) [file pone.0026590.s002.ppt]

## Slide 1
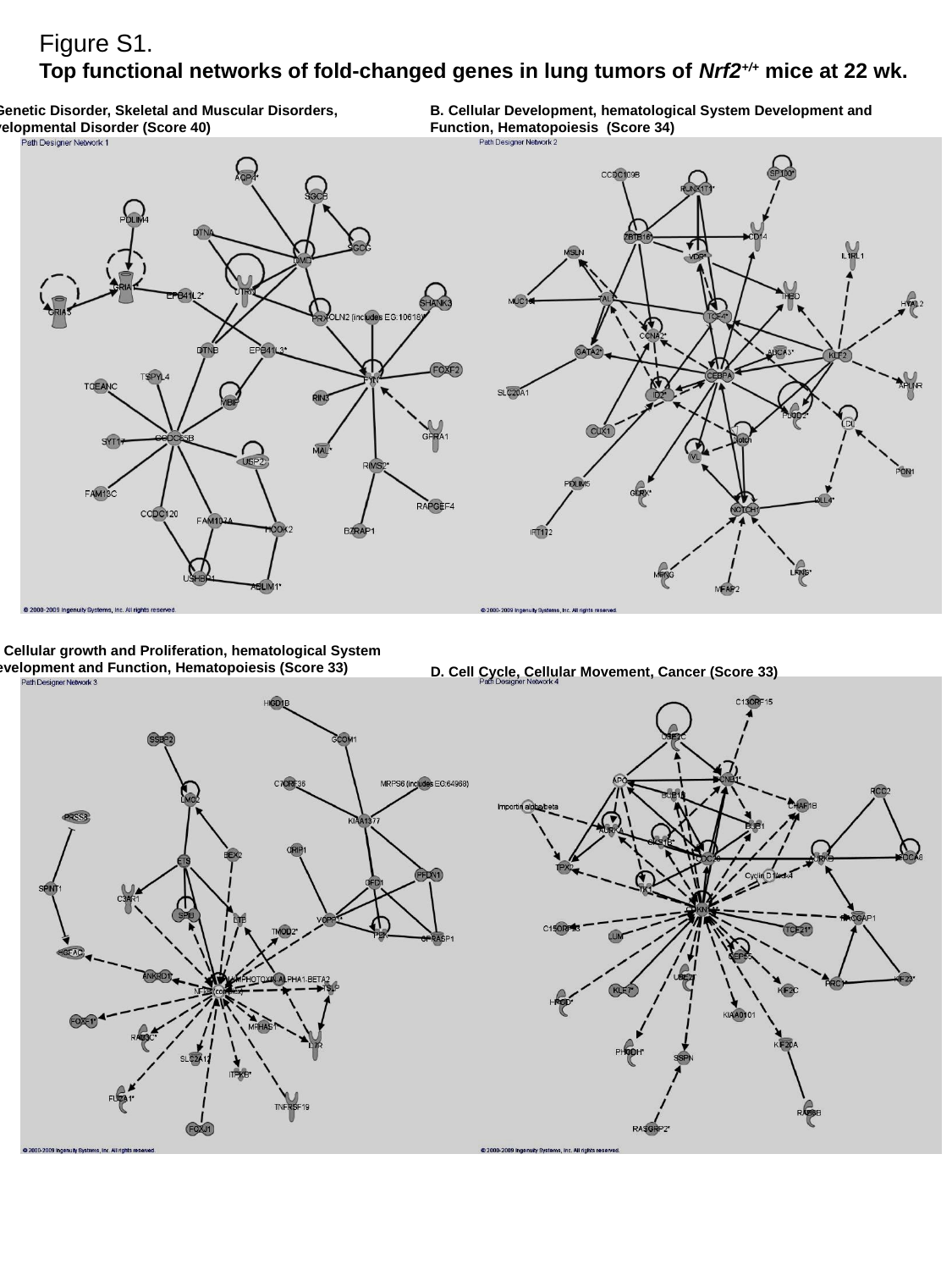

Figure S1.
Top functional networks of fold-changed genes in lung tumors of Nrf2+/+ mice at 22 wk.
A. Genetic Disorder, Skeletal and Muscular Disorders,
Developmental Disorder (Score 40)
B. Cellular Development, hematological System Development and
Function, Hematopoiesis (Score 34)
C. Cellular growth and Proliferation, hematological System
Development and Function, Hematopoiesis (Score 33)
D. Cell Cycle, Cellular Movement, Cancer (Score 33)

## Slide 2
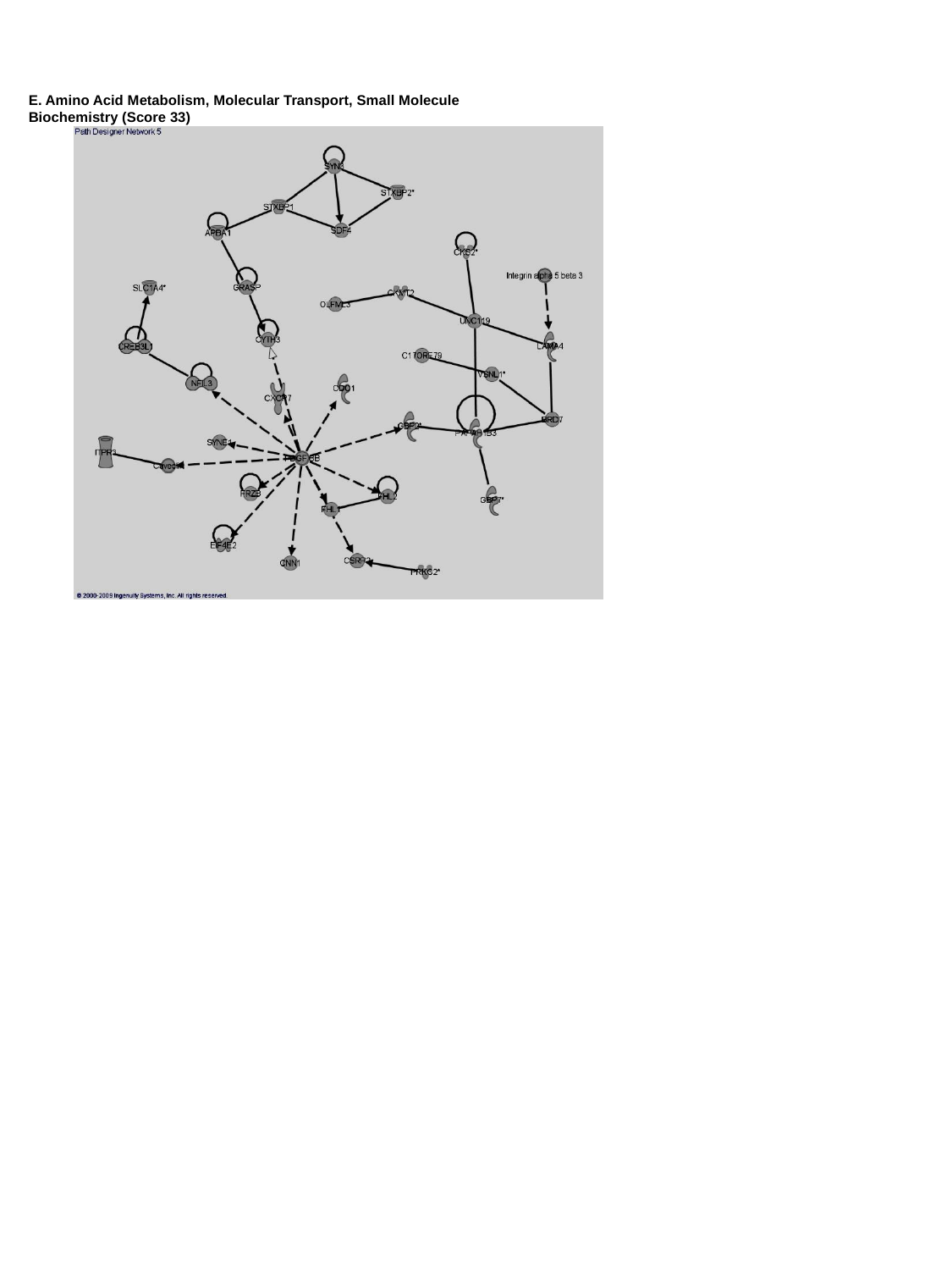

E. Amino Acid Metabolism, Molecular Transport, Small Molecule
Biochemistry (Score 33)
