# Supplementary material for: Targeted Deletion of Nrf2 Reduces Urethane-Induced Lung Tumor Development in Mice
Source: PLoS One. 2011 Oct 21;6(10):e26590. doi: 10.1371/journal.pone.0026590 (PMC3198791; doi:10.1371/journal.pone.0026590)
Supplement: Figure S3 — Top functional networks of Nrf2-dependent genes changed during urethane-induced tumorigenesis. (A) Ingenuity Pathway Analysis (IPA) generated a key network (Score 54) of cell-to-cell signaling and interaction-connective tissue development and function-ophthalmic disease with Nrf2-dependently regulated genes in pre-/early-neoplastic microenvironment (12 wk), in which genes encoding matrix metalloproteinase 2 (Mmp2) and D site albumin promoter binding protein (Dbp) were mapped as core molecules. (B) Nrf2-dependent lung tumor genes were highly associated in the networks of cell cycle-cancer-connective tissue development and function (Score 43, e.g., cyclin D1, Ccnd1; E2F transcription factor 3, E2f3), cell-to-cell signaling and interaction-tissue development-cell function (Score 38, e.g., chemokine (C-X-C motif) ligand 1, Cxcl1; integrin alpha 4, Itga4), and tumor morphology-cancer-dermatological disease and conditions (Score 38, e.g., G protein-coupled 56, Gpr56; glutathione-S-transferase, alpha 3, Gsta3) as generated by IPA. (C) Nrf2-dependently modulated genes in common (n = 21 genes) in early-neoplastic microenvironment (12 wk) and in lung tumors (22 wk) were functionally associated in cancer-cell cycle-cell death network (Score 23). They included genes encoding CD34, UGT1a1, fetuin beta (Fetub), G protein-coupled receptor 137B (Gpr137b), ATP binding cassette, subfamily C, member 4 (Abcc4), phosphogluconate dehydrogenase (Pgd), etc. (.ppt). (PPT) [file pone.0026590.s003.ppt]

## Slide 1
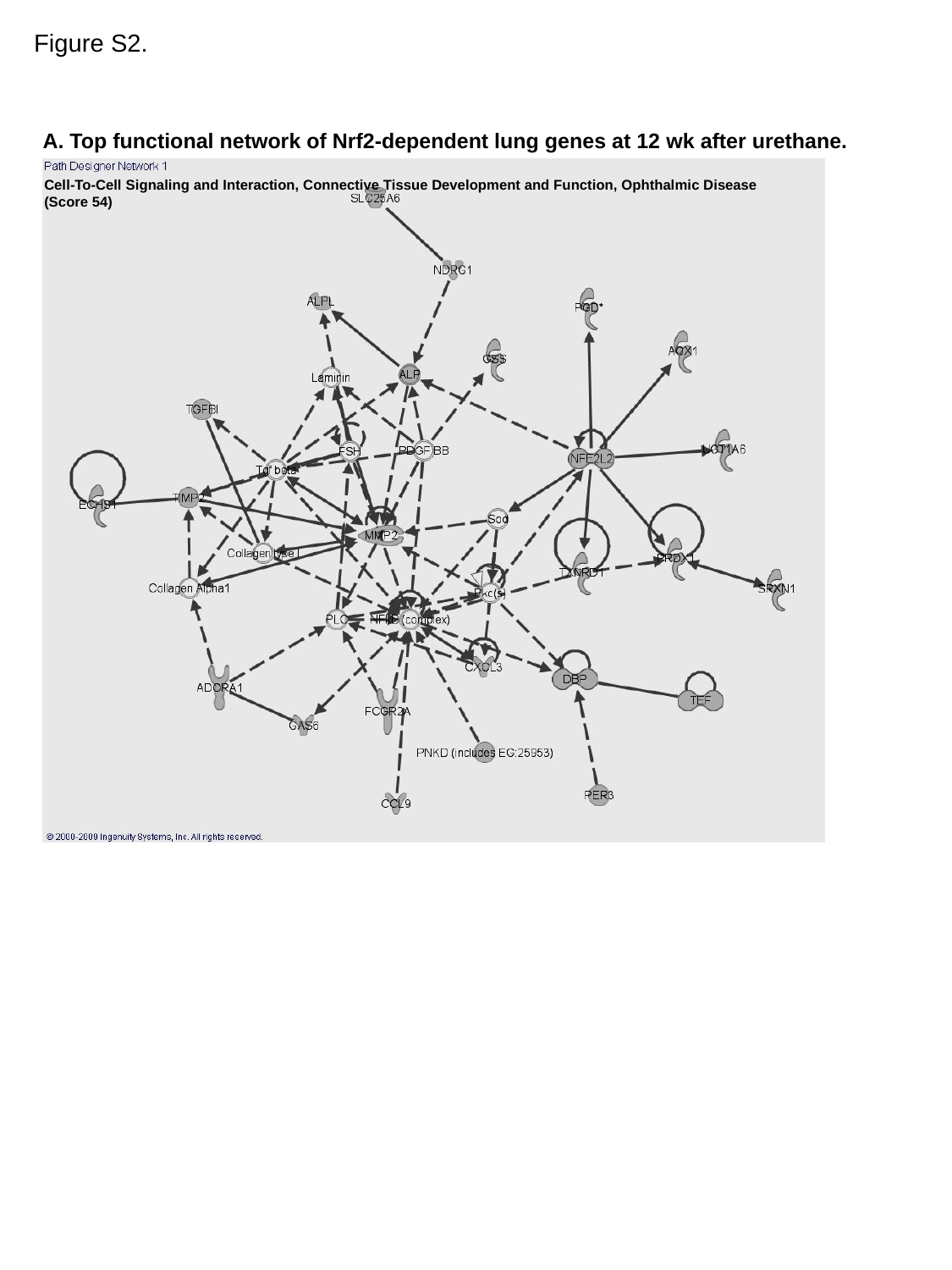

Figure S2.
A. Top functional network of Nrf2-dependent lung genes at 12 wk after urethane.
Cell-To-Cell Signaling and Interaction, Connective Tissue Development and Function, Ophthalmic Disease
(Score 54)

## Slide 2
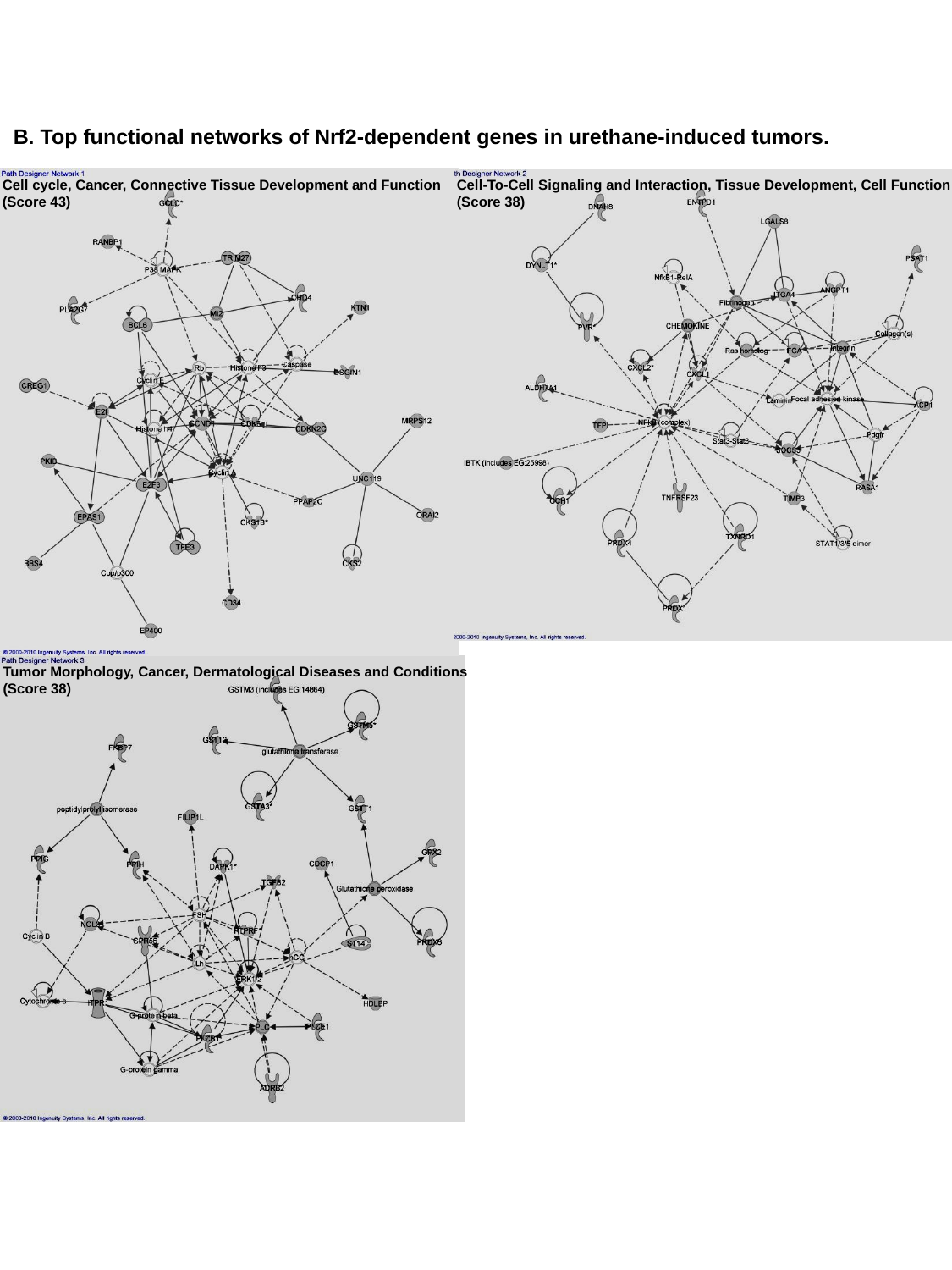

B. Top functional networks of Nrf2-dependent genes in urethane-induced tumors.
Cell cycle, Cancer, Connective Tissue Development and Function
(Score 43)
Cell-To-Cell Signaling and Interaction, Tissue Development, Cell Function
(Score 38)
Tumor Morphology, Cancer, Dermatological Diseases and Conditions
(Score 38)

## Slide 3
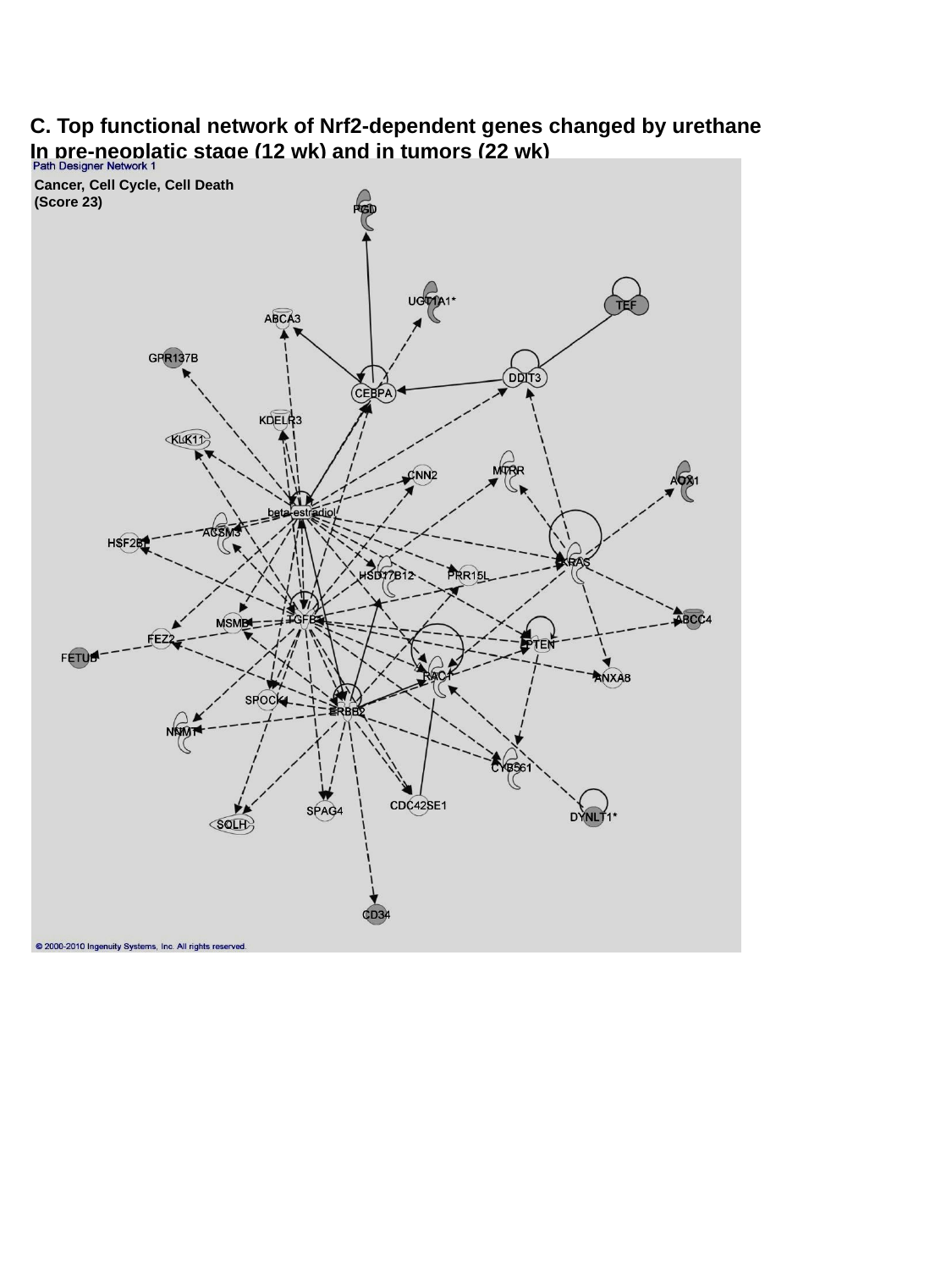

C. Top functional network of Nrf2-dependent genes changed by urethane
In pre-neoplatic stage (12 wk) and in tumors (22 wk)
Cancer, Cell Cycle, Cell Death
(Score 23)
