# Supplementary material for: Targeted Deletion of Nrf2 Reduces Urethane-Induced Lung Tumor Development in Mice
Source: PLoS One. 2011 Oct 21;6(10):e26590. doi: 10.1371/journal.pone.0026590 (PMC3198791; doi:10.1371/journal.pone.0026590)
Supplement: Table S1 — Representative lung genes significantly changed by urethane at 12 wk in Nrf2+/+ mice (n = 6519, p <0.05). (DOC) [file pone.0026590.s005.doc]

**Table S1.** Representative lung genes significantly changed by urethane at 12 wk in *Nrf2^+/+^* mice (n=6519, *p*<0.05).

| **GI Accession** | **Gene Symbol** | **Description** | | **Fold Change**  **(vs. saline)** |
| --- | --- | --- | --- | --- |
| **Cell cycle and apoptosis** | | | | |
| BG065754 | *Ccng1* | cyclin G1 | | 3.0 |
| AK007630 | *Cdkn1a* | cyclin-dependent kinase inhibitor 1A (P21) | | 3.1 |
| NM_028760 | *Cep55* | centrosomal protein 55 | | 2.1 |
| **Cell growth and organ development** | | | | |
| NM_007950 | *Ereg* | epiregulin | | 2.0 |
| NM_009704 | *Areg* | amphiregulin | | 2.5 |
| AV246296 | *Eda2r* | ectodysplasin A2 isoform receptor | | 3.0 |
| NM_028618 | *Dmkn* | dermokine | | 2.8 |
| **Transport** | | | | |
| NM_011867 | *Slc26a4* | solute carrier family 26, member 4 | | 2.1 |
| AK006096 | *Mfsd2* | major facilitator superfamily domain containing 2 | | 2.4 |
| NM_017399 | *Fabp1* | fatty acid binding protein 1, liver | | 2.1 |
| BF468072 | *Slc12a5* | solute carrier family 12, member 5 | | -2.6 |
| **Immune response** | | | | |
| NM_021443 | *Ccl8* | chemokine (C-C motif) ligand 8 | | 2.3 |
| NM_008599 | *Cxcl9* | chemokine (C-X-C motif) ligand 9 | | 3.4 |
| NM_009404 | *Tnfsf9* | tumor necrosis factor (ligand) superfamily, member 9 | | 3.2 |
| NM_009141 | *Cxcl5* | chemokine (C-X-C motif) ligand 5 | | -2.9 |
| **Cell adhesion** | | | | |
| NM_054072 | *Pcdha1* | protocadherin alpha 1 | | -2.6 |
| NM_007727 | *Cntn1* | contactin 1 | | -2.2 |
| **Proteolysis** |  |  |  |  |
| BC019135 | *Mmp12* | matrix metallopeptidase 12 | | 3.0 |
| AB032200 | *Gzmk* | granzyme K | | 2.2 |
| **Metabolism** | | | | |
| NM_007994 | *Fbp2* | fructose bisphosphatase 2 | | 2.2 |
| NM_009127 | *Scd1* | stearoyl-Coenzyme A desaturase 1 | | 2.3 |
| NM_009705 | *Arg2* | arginase type II | | 1.9 |
| **Microtubule and structure** | | | | |
| NM_019976 | *Psrc1* | proline/serine-rich coiled-coil 1 | | 4 |
| AV337593 | *Mtap2* | microtubule-associated protein 2 | | 2 |
| NM_177757 | *Kif26b* | kinesin family member 26B | | -3.0 |
| BB357580 | *Cttnbp2* | cortactin binding protein 2 | | -2.2 |
| **Stress and defense** | | | | |
| NM_009676 | *Aox1* | aldehyde oxidase 1 | | 1.5 |
| NM_172759 | *Ces5* | carboxylesterase 5 | | 2.0 |
