# Supplementary material for: Targeted Deletion of Nrf2 Reduces Urethane-Induced Lung Tumor Development in Mice
Source: PLoS One. 2011 Oct 21;6(10):e26590. doi: 10.1371/journal.pone.0026590 (PMC3198791; doi:10.1371/journal.pone.0026590)
Supplement: Table S2 — Top functional networks and involved genes significantly ( p <0.05) changed in uninvolved tissues and in tumors of Nrf2+/+ mice at 22 wk. (DOC) [file pone.0026590.s006.doc]

**Table S2.** Top functional networks and involved genes significantly (*p*<0.05) changed in uninvolved tissues and in tumors of *Nrf2^+/+^* mice at 22 wk.

| **Top functional network** | ***Score*** | **Representative focused genes and fold changes (*vs.* saline)** | | | |
| --- | --- | --- | --- | --- | --- |
| ***In Uninvolved Tissues (ANOVA 1564 genes, 2-fold 27 genes)*** | | | | | |
| Cell-To-Cell Signaling and Interaction, Cell Death, Nervous System Development and Function | 30 | NM_019976 | *Psrc1* | proline/serine-rich coiled-coil 1 | 2.44 |
|  |  | NM_020333 | *Slc12a5* | solute carrier family 12, member 5 | -2.75 |
|  |  | NM_007897 | *Ebf1* | early B-cell factor 1 | -2.16 |
|  |  | NM_001003671 | *Pcdha1* | protocadherin alpha 1 | -2.01 |
|  |  | NM_080639 | *Timp4* | tissue inhibitor of metalloproteinase 4 | -2.23 |
| ***In Tumors (ANOVA 8683 genes, 2-fold 3461 genes)*** | | | | | |
| Genetic Disorder, Skeletal and Muscular Disorders, Developmental Disorder  (Supp. Fig. 1-A) | 40 | NM_009700 | *Aqp4* | aquaporin 4 | -9.25 |
|  |  | NM_010225 | *Foxf2* | forkhead box F2 | -4.45 |
|  |  | NM_001122892 | *Fyn* | Fyn proto-oncogene | -2.49 |
|  |  | NM_010762 | *Mal* | myelin and lymphocyte protein | 3.63 |
|  |  | NM_019412 | *Prx* | periaxin | -7.41 |
|  |  | NM_011890 | *Sgcb* | sarcoglycan, beta | -2.48 |
|  |  | NM_021423 | *Shank3* | SH3/ankyrin domain gene 3 | -5.38 |
|  |  | NM_001007577 | *Tceanc* | transcription elongation factor A (SII) N-terminal and central domain containing | 6.15 |
|  |  | NM_009443 | *Tgoln1* | trans-golgi network protein | 3.46 |
| Cellular and Hematological System Development and Function, Hepatopoiesis  (Supp. Fig. 1-B) | 34 | NM_009841 | *Cd14* | CD14 antigen | 3.99 |
|  |  | NM_019454 | *Dll4* | delta-like 4 (Drosophila) | -2.85 |
|  |  | NM_008090 | *Gata2* | GATA binding protein 2 | -5.15 |
|  |  | NM_010496 | *Id2* | inhibitor of DNA binding 2 | 4.58 |
|  |  | NM_008412 | *Ivl* | involucrin | 9.51 |
|  |  | NM_018857 | *Msln* | mesothelin | -3.72 |
|  |  | NM_011134 | *Pon1* | paraoxonase 1 | -21.4 |
|  |  | NM_011527 | *Tal1* | T-cell acute lymphocytic leukemia 1 | -3.40 |
|  |  | NM_009378 | *Thbd* | thrombomodulin | -6.52 |
|  |  | NM_001033324 | *Zbtb16* | zinc finger and BTB domain containing 16 | -6.52 |
| Cellular Growth and Proliferation, Hematological System Development and Function, Hematopoiesis  (Supp. Fig. 1-C) | 33 | NM_013468 | *Ankrd1* | ankyrin repeat domain 1 | -10.86 |
|  |  | NM_008240 | *Foxj1* | forkhead box J1 | -5.20 |
|  |  | NM_001033208 | *Gcom1* | GRINL1A complex locus | -7.32 |
|  |  | NM_008372 | *Il7r* | interleukin 7 receptor | -2.44 |
|  |  | NM_023852 | *Rab3c* | RAB3C, member RAS oncogene family | 2.76 |
|  |  | NM_178934 | *Slc2a12* | solute carrier family 2 (facilitated glucose transporter), member 12 | -5.27 |
|  |  | NM_001038710 | *Tmod2* | tropomodulin 2 | -4.84 |
|  |  | NM_013869 | *Tnfrsf19* | tnf receptor superfamily, member 19 | -7.68 |
| Cell Cycle, Cellular Movement, Cancer  (Supp. Fig. 1-D) | 33 | NM_172301 | *Ccnb1* | cyclin B1 | 3.82 |
|  |  | NM_023223 | *Cdc20* | cell division cycle 20 homolog | 4.02 |
|  |  | NM_028760 | *Cep55* | centrosomal protein 55 | 4.92 |
|  |  | NM_016904 | *Cks1b* | CDC28 protein kinase 1b | 4.35 |
|  |  | NM_008278 | *Hpgd* | hydroxyprostaglandin dehydrogenase 15 | -6.43 |
|  |  | NM_033563 | *Klf7* | Kruppel-like factor 7 (ubiquitous) | -3.08 |
|  |  | NM_173781 | *Rab6b* | RAB6B, member RAS oncogene family | -4.8 |
|  |  | NM_011545 | *Tcf21* | transcription factor 21 | -5.08 |
|  |  | NM_026785 | *Ube2c* | ubiquitin-conjugating enzyme E2C | 3.52 |
| Amino Acid Metabolism, Molecular Transport, Small Molecule Biochemistry  (Supp. Fig. 1-E) | 33 | NM_033037 | *Cdo1* | cysteine dioxygenase 1, cytosolic | -7.67 |
|  |  | NM_198415 | *Ckmt2* | creatine kinase, mitochondrial 2 | -16.65 |
|  |  | NM_009922 | *Cnn1* | calponin 1 | -16.29 |
|  |  | NM_011182 | *Cyth3* | cytohesin 3 | -4.47 |
|  |  | NM_008776 | *Pafah1b3* | platelet-activating factor acetylhydrolase, isoform 1b, subunit 3 | 3.56 |
|  |  | NM_008926 | *Prkg2* | protein kinase, cGMP-dependent, type II | -7.27 |
| Cell Morphology, Renal and Urological System Development and Function, Cellular Compromise | 31 | NM_001014399 | *Abi3bp* | ABI gene family, member 3 binding protein | -18.0 |
|  |  | NM_011606 | *Clec3b* | C-type lectin domain family 3, member b | -3.96 |
|  |  | NM_033373 | *Krt23* | keratin 23 | 6.05 |
|  |  | NM_001136069 | *Ldha* | lactate dehydrogenase A | 2.42 |
