# Supplementary material for: Targeted Deletion of Nrf2 Reduces Urethane-Induced Lung Tumor Development in Mice
Source: PLoS One. 2011 Oct 21;6(10):e26590. doi: 10.1371/journal.pone.0026590 (PMC3198791; doi:10.1371/journal.pone.0026590)
Supplement: Methods S1 — (DOC) [file pone.0026590.s010.doc]

**Methods S1**

***Lung fixation, tumor analysis, histopathology and immunohistopathology.*** For tumor enumeration at 22 wk post injection, lungs were isolated, inflated with Tellyesniczky's fixative and fixed for 48 h in the same fixative (1). Lung lobes were dissected and the numbers of tumors were counted using Olympus SZX7 light microscopy (Olympus, Center Valley, PA). Tumor size was also evaluated in the same lungs using a digital caliper (Mitutoyo Co., Japan). Sizes over 200 μm were considered as microadenomas and analyzed for light microscoscopic evaluation. At 12 wk post injection, 10% neutral buffered formalin-fixed whole lungs were sectioned serially from the distal edge, and microadenomas and hyperplasias were histologically evaluated in hematoxylin and eosin (H&E)-stained lung sections from every 10, evenly distributed, sections. To examine the changes in airway epithelial mucous production and secretion during the inflammatory phase, lung sections from 9 wk post injection were stained with AB/PAS reagent to detect acidic and neutral mucosubstances. Immunohistological staining was done using specific antibodies against PCNA (sc-56, Santa Cruz Biotechnology, Santa Cruz, CA), Ki67 (ab15580, Abcam, Inc., Cambridge, MA), and Nrf2 (sc-722, Santa Cruz Biotechnology) following previously published procedures (2). Apoptotic cell death was determined on lung tissue sections *in situ* by assessing cellular DNA fragmentation using a TUNEL kit (Promega, Madison, WI) as described previously (3). The numbers of TUNEL-stained nuclei in proximal sections (G5) were counted and normalized to the area of lung section surface using AxioVision AC V.4.5 digital image processing software and a AxioCamMR3 camera connected to a Axioskop40 light microscope (Carl Zeiss Microimaging, Inc., Thornwood, NY).

***Characterization of Microarray Data.*** GeneSpring Expression Analysis (Agilent Technologies, Inc., Santa Clara, CA) was used for statistical analyses and characterization of data. Intensity of each gene on GeneChip triplicates per group were normalized to the mean intensity of the same gene in time-matched *Nrf2^+/+^* saline control group, and these relative expression ratios were used for all statistical comparisons. Statistical analysis of array data was done in three ways. First, urethane-induced changes in lung gene expression files in wild-type mice were determined at 12 wk (saline vs. urethane) and 22 wk (saline vs. UN vs. tumor) after urethane treatment was initiated. 6519 gene transcripts were significantly changed by urethane (t-test, p<0.05) at 12 wk; among these transcripts, 35 were increased and 17 were decreased 2-fold or more relative to saline controls. At 22 wk, 1-way analysis of variance (ANOVA, *p*<0.05) identified 8683 gene transcripts that were significantly changed by urethane. Most of these were significantly changed relative to saline controls only in tumor tissues (7197 genes) while 78 were significantly changed only in UN tissues (all <2-fold changed). Transcripts that were changed greater than 2-fold relative to saline control (3461 from saline vs. tumor, 27 from saline vs. UN) were filtered for further analysis. In the second analysis, 2-way ANOVA identified 118 gene transcripts that were differentially expressed (*p*<0.05) between *Nrf2^+/+^* and *Nrf2^-/-^* mice at 12 wk after urethane, and these were further profiled by k-means clustering. In the final analysis, data from saline control, UN, and tumor tissues of *Nrf2^+/+^* and *Nrf2^-/-^* mice at 22 wk were analyzed by 2-way ANOVA (*p*<0.01) followed by Benjamini and Hochberg False Discovery Rate test for the multiple comparisons, which elucidated Nrf2-dependent gene transcripts that were differentially expressed between saline and tumor (376 genes), saline and UN (11 genes), or UN and tumor (154 genes). Clustering analysis classified 154 Nrf2-dependent transcripts that were different between saline and tumor into 4 sets of similar expression kinetic patterns. Ingenuity Pathway Analysis (IPA) software (www.ingenuity.com, Ingenuity Systems, Inc., Redwood City, CA), a computated algorhythmic finding tool based on scientifically demonstrated knowledge, was applied to identify biological insights to these filtered genes. IPA identified potentially significant functional connections and mechanistic pathways through which urethane causes lung tumors and the role of Nrf2 in the process. The raw microarray data have been deposited in NIEHS Chemical Effects in Biological Systems (CEBS, http://cebs.niehs.nih.gov, accession #: 005-00003-0080-000-6).

***Quantitative reverse transcriptase-polymerase chain reaction (qRT-PCR).*** An aliquot (1 μg) of total lung RNA was reverse transcribed as previously published (4) and gene specific primers used in quantitative RT-PCR reactions, similar to previous publications (5). cDNA equivalent to 20-50 ng of RNA was amplified in a 20 μl volume containing gene-specific primers labeled with FAM or SYBR Green dyes (TaqMan qPCR gene Expression Assays, Applied Biosystems; Real Time Primers, LLC, Elkins Park, PA) and proper 2X Master Mix (Applied Biosystems, Foster City, CA) using an ABI Prism 7700 Sequence Detection System (Applied Biosystems). The relative quantification of gene expression was calculated from the threshold cycle (C_T_) values for each sample and normalized in relation to the expression of 18s rRNA using the comparative C_T_ method.

**References**

1. Bauer AK, Dixon D, DeGraff LM, Cho HY, Walker CR, Malkinson AM, Kleeberger SR. Toll-like receptor 4 in butylated hydroxytoluene-induced mouse pulmonary inflammation and tumorigenesis. *J Natl Cancer Inst* 2005;97:1778-1781.

2. Cho HY, Morgan DL, Bauer AK, Kleeberger SR. Signal transduction pathways of tumor necrosis factor--mediated lung injury induced by ozone in mice. *American journal of respiratory and critical care medicine* 2007;175:829-839.

3. Cho HY, Gladwell W, Wang X, Chorley B, Bell D, Reddy SP, Kleeberger SR. Nrf2-regulated ppar{gamma} expression is critical to protection against acute lung injury in mice. *American journal of respiratory and critical care medicine* 2010;182:170-182.

4. Cho HY, Jedlicka AE, Reddy SP, Kensler TW, Yamamoto M, Zhang LY, Kleeberger SR. Role of nrf2 in protection against hyperoxic lung injury in mice. *Am J Respir Cell Mol Biol* 2002;26:175-182.

5. Bauer AK, Fostel J, Degraff LM, Rondini EA, Walker C, Grissom SF, Foley J, Kleeberger SR. Transcriptomic analysis of pathways regulated by toll-like receptor 4 in a murine model of chronic pulmonary inflammation and carcinogenesis. *Mol Cancer* 2009;8:107.
